# Ultra**-**small Pyropheophorbide**-**a Nanodots for Near**-**infrared Fluorescence/Photoacoustic Imaging-guided Photodynamic Therapy

**DOI:** 10.7150/thno.35735

**Published:** 2020-01-01

**Authors:** Kittipan Siwawannapong, Rui Zhang, Huali Lei, Qiutong Jin, Wantao Tang, Ziliang Dong, Rung-Yi Lai, Zhuang Liu, Anyanee Kamkaew, Liang Cheng

**Affiliations:** 1 School of Chemistry, Institute of Science and Center of Excellent-Advanced Functional Materials, Suranaree University of Technology, Nakhon Ratchasima 30000, Thailand.; 2 Institute of Functional Nano & Soft Materials (FUNSOM), Collaborative Innovation Center of Suzhou Nano Science and Technology, Soochow University, Suzhou, Jiangsu 215123, China.

**Keywords:** Pa-PEG ultra-small nanoparticles, Dual-modal imaging, Photodynamic therapy, Renal clearance, toxicity

## Abstract

**Rationale**: Nanoparticles (NPs) that are rapidly eliminated from the body offer great potential in clinical test. Renal excretion of small particles is preferable over other clearance pathways to minimize potential toxicity. Thus, there is a significant demand to prepare ultra-small theranostic agents with renal clearance behaviors.

**Method**: In this work, we report a facile method to prepare NPs with ultra-small size that show renal clearable behavior for imaging-guided photodynamic therapy (PDT). Pyropheophorbide-a (Pa), a deep red photosensitizer was functionalized with polyethylene glycol (PEG) to obtain Pa-PEG. The prepared NPs formed ultra-small nanodots in aqueous solution and showed red-shifted absorbance that enabling efficient singlet oxygen generation upon light irradiation.

**Results**: *In vitro* studies revealed good photodynamic therapy (PDT) effect of these Pa-PEG nanodots. Most of the cancer cells incubated with Pa-PEG nanodots were destroyed after being exposed to the irradiated light. Utilizing the optical properties of such Pa-PEG nanodots, *in vivo* photoacoustic (PA) and fluorescence (FL) imaging techniques were used to assess the optimal time for PDT treatment after intravenous (i.v.) injection of the nanodots. As monitored by the PA/FL dual-modal imaging, the nanodots could accumulate at the tumor site and reach the maximum concentration at 8 h post injection. Finally, the tumors on mice treated with Pa-PEG nanodots were effectively inhibited by PDT treatment. Moreover, Pa-PEG nanodots showed high PA/FL signals in kidneys implying these ultra-small nanodots could be excreted out of the body *via* renal clearance.

**Conclusion**: We demonstrated the excellent properties of Pa-PEG nanodots that can be an *in vivo* imaging-guided PDT agent with renal clearable behavior for potential future clinical translation.

## 1. Introduction

Photodynamic therapy **(**PDT**)** has emerged as a relatively new treatment method with high potential for cancer therapy. PDT is considered as a noninvasive treatment with less side effect compared to conventional cancer treatments such as radiation and chemo**-**therapies 1-4**.** To date, researchers are still developing strategies to improve the effectiveness of PDT and expand it to treat various types of cancer**.** One way to enhance PDT efficacy is to develop near**-**infrared **(**NIR**)** responsive photosensitizers** (**PSs**)** that are able to generate reactive oxygen species **(**ROS**)** such as singlet oxygen **(**^1^O_2_**)** to destroy cancerous cells under NIR light exposure**.** Moreover, some PSs have been used as imaging agents to monitor biodistribution of materials as guidance for PDT 5, 6.

Porphyrin and its derivatives are widely used as PSs in PDT since they are low-toxic, possess NIR absorption, and can produce singlet oxygen, a key cytotoxic agent for tumor devastation, after light irradiation 7-11**.** Chlorophyll**-**a and its derivatives are one family containing a large π conjugation system of porphyrin structure, which provides strong absorption in the 300**-**700 nm region**.** Major disadvantages of chlorophyll**-**a derivatives and other porphyrins are their naturally high hydrophobicity and have poor tumor**'**s selectivity 12, 13**.** To tackle these problems, hydrophilic bioconjugated molecules, such as polymers, dendrimers, peptides or lipids are introduced to improve the hydrophilicity of those molecules 1, 7, 14.

Based on enhanced permeability and retention **(**EPR**)** effect, nanoparticles loaded PSs may be trapped within solid tumors after intravenous injection 15**.** Hence, modified**-**nanomaterials containing both photodynamic therapeutic and imaging capabilities have received much attention in oncology for cancer diagnosis and therapy 16-18**.** Pyropheophorbide**-**a **(**Pa**)**, a molecule derived from chlorophyll**-**a, was recently used to conjugate with nanomaterials as effective PDT agents *in vitro* and *in vivo* 19-23, 24**.** Rapozzi and co-workers reported the effect of PEGylation on the photosensitizer (pheophorbide**-**a) biodistribution 25**.** The results showed that the nanomaterials **(**mPEG**-**Pba**)** could be distributed to a whole body with higher amount of photosensitizer in the tumor compared to free Pba**.** PEGylation of photosensitizers **(**Pba and Ce 6**)** exhibited efficient intracellular uptake and phototoxicity *in vitro* for cancer treatment**.** Yet, *in vivo* studies were not reported in these works 26-29**.** However, the relatively large diameter of these nanomaterials led to the prolonged retention in reticuloendothelial system **(**RES**)** organs **(**liver and spleen**)**, resulting in poor clearance from the body due to the sluggish excretion *via* hepatocyte 30, 31**.** Hence, the long**-**retention period of those nanomaterials can cause long**-**term effects **(**e**.**g**.** potential toxicity**)** that restrict their clinical translation**.** Notably, ultra**-**small nanoparticles with size less than 6**-**8 nm can pass through the glomerular capillary of kidney filtration to enable faster elimination from the body *via* renal partway compared to hepatobiliary excretion 32-35**.** Moreover, there are some reported nanoparticles with size between 1**-**20 nm containing negative charge on the surface tend to have renal excretable properties 36**.** To balance tumor retention ability and renal eliminable behavior for reducing long**-**term effects, it is a great interest to explore renal**-**clearable nanomaterials containing imaging**-**guided and therapeutic properties for cancer therapy.

Recently, photoacoustic **(**PA**)** imaging has developed as a new method for imaging**-**guided therapy, based on NIR excitation and ultrasound signal emission 37-41**.** Moreover, this technique can provide deeper tissue penetration due to the far optical absorption window **(**700**-**900 nm**)**, and high spatial resolution**.** Compared to PA, fluorescence **(**FL**)** imaging offers higher resolution and greater sensitivity, whereas it has poor spatial resolution due to the limitation of light penetration ability 42-44**.** Therefore, combining FL and PA imaging modalities in a single particle may overcome the limitation of these two imaging techniques, which enhance imaging resolution and sensitivity for tracking the accumulation of nanomaterials 45.

Herein, we reported Pa**-**PEG nanodots for the first time use as PA**/**FL imaging**-**guided PDT with renal clearance properties**.** Based on our previous study 46, terminated **-**NH_2_ PEG** (**MW 5k**)** was one of the good candidates for effective renal clearance with promising tumor accumulation**.** Thus, the Pa**-**PEG nanodots were successfully prepared *via* an amide coupling reaction between terminated **-**NH_2_ PEG and Pa**-**COOH to generate ultra**-**small nanodots with ~2 nm in TEM size**.** The synthesized nanodots showed good stability in various physiological solutions**.** All *in vitro* results demonstrated Pa**-**PEG nanodots have a remarkable potential to induce cytotoxicity against cancerous cells upon irradiation with a red LED lamp**.** Guidance by PA**/**FL dual imaging techniques, the optimal time for PDT treatment was suggested to be 8 h after intravenous **(**i**.**v**.)** injection**.** *In vivo* PDT conducted in 4T1 tumor**-**bearing mice exhibited great therapeutic efficacy under light irradiation with renal excretable behavior and no long**-**term side effects.

## 2. Experimental Section

**Materials.** Pyropheophorbide-a **(**Pa**)** was purchased from Frontier Scientific**.** Methoxypolyethylene glycol amine 5 kDa **(**mPEG**-**NH_2_**)** was purchased from Biomatrik Co**.**, Ltd**. (**Jiaxing, China**)**, 1**-**ethyl**-**3**-(**3**-**dimethylaminopropyl**)** carbodiimide **(**EDC**)** was obtained from Sigma**-**Aldrich**.** Deionized** (**DI**)** water was purified from the Milli**-**Q purification system.

**Synthesis of Pa-PEG Nanodots and Purification.** Pa**-**PEG nanodots were synthesized by a facile method similar to the protocol previously reported 46**.** Briefly, Pa **(**10 µmol**)** and EDC **(**30 µmol**)** were mixed in 1 mL dimethyl sulfoxide **(**DMSO**)** and stirred for 1 h at 25 ºC to activate a carboxylic group on Pa**.** After that, the activated**-**Pa was added wisely into DMSO solution containing mPEG**-**NH_2_** (**9**.**5 μmol**)** and the solution was continued stirring for 24 h at 25 ºC**.** Excess amounts of EDC, unreacted Pa, and DMSO were removed by dialysis for 24 h against DI water using a dialysis bag with molecular weight cut off **(**MWCO**)** 8,000**-**14,000 Da**.** Finally, the final product was obtained by filtration using Millipore filter** (**10 kDa MWCO**)** and washed three times with DI water**.** The resulting Pa**-**PEG nanodots were re**-**dispersed in 3 mL DI water and stored at 4 °C for future use.

**Characterization.** TEM images of Pa**-**PEG nanodots were conducted using a Tecnai F20 transmission electron microscope **(**FEI**).** Hydrodynamic sizes **(**HDs**)** were determined by a Zetasizer Nano Z **(**Malvern**).** Absorption spectra were collected from a PerkinElmer Lambda 750 UV**-**Vis**-**NIR spectrometer**.** Fluorescence spectra were obtained from Horiba FluoroMax 4 spectrometer**.** ^1^H**-**NMR spectra were recorded on a 600 MHz NMR spectrometer** (**DDZ**-**600**).** Matrix Assisted Laser Desorption**/**Ionization**-**Time of Flight Mass Spectrometry **(**MALDI**-**TOF**)** data was obtained from ultrafleXtremeTM, which was made by Bruker Daltonics, Germany.

**Singlet Oxygen Detection.** Singlet oxygen production was detected according to the previously reported protocol 46**.** Briefly, singlet oxygen sensor green **(**SOSG**)** 10 μL** (**0**.**5 mM**)** was added into 2 mL of Pa**-**PEG nanodots solution containing 30 μM of Pa**.** Then, the solution was exposed to a red LED lamp** (**600~700 nm**)** at a power density of 10 mW cm**^-^**^2^ for 5, 10, 20, 30, and 60 min**.** Free Pa solution in DMSO**/**water at the same Pa concentration of Pa**-**PEG nanodots and DI water were also carried out as controls using the same method**.** Finally, fluorescent intensities of SOSG were measured at an excitation of 494 nm.

**Singlet Oxygen Quantum Yield.** Singlet oxygen quantum yield **(**Ф**_∆_)** of free Pa and Pa**-**PEG nanodots were carried out *via* an indirect method, using zinc phthalocyanine** (**ZnPc**)** and 1,3**-**diphenylisobenzofuran** (**DPBF**)** as a standard and singlet oxygen scavenger, respectively 47**.** In brief, 20 μL of 10 μM DPBF was dropped into 3 mL of Pa**-**PEG solution containing 2 μM of Pa in DMSO**.** Then, the mixed solutions were exposed to a red LED lamp at a power density of 10 mW cm**^-^**^2^ for 0, 1, 2, 3, and 4 seconds**.** Absorption intensities of DPBF at 418 nm were determined to quantify the singlet oxygen consuming rates using a plate reader** (**Epoch Microplate Spectrophotometer**).** To check photostability of material under the presence of singlet oxygen, the absorbance of Pa**-**PEG nanodots at 660 nm was also recorded at the same time points**.** ZnPc and free Pa were also examined as the standard and control at the same concentration using the same method**.** Then, the Ф**_∆_**values were calculated following equation** (**1**):**

Ф**∆ =**
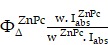
** (**1**)**

where **(**
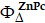
**)** is the singlet oxygen quantum yield for ZnPc **(**Ф**_∆_ =** 0**.**67 in DMSO**) 48**; **w** and **w^ZnPc^** are the DPBF photobleaching rates in the presence of Pa**-**PEG nanodots and ZnPc, respectively; **I_abs_** and 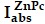 are the light absorption values for Pa**-**PEG nanodots **(**λ** =** 660 nm**)** and ZnPc **(**λ** =** 660 nm**)**, respectively.

**Cell Culture and *In vitro* Experiments.** 4T1 cells **(**murine breast cancer**)** were cultured in RPMI**-**1640 medium supplementing 10** %** fetal bovine serum **(**FBS**)**, 1** %** penicillin**/**streptomycin, and 2 mL of glutamine 200 mM**.** The cells were kept at 37 °C under a humidified atmosphere containing 5** %** CO_2_.

*In vitro PDT***:** 4T1 cells were seeded at a density of 1 x 10^4^ cells**/**well into 96**-**well plates and incubated with the Pa**-**PEG nanodots at various concentrations** (**0**-**40 µM**)** for 24 h**.** The cells from the experimental group were irradiated by a red LED lamp at a power density of 10 mW cm**^-2^** for 30 and 60 min, whereas the control groups were kept in the dark**.** Subsequently, all cells were cultured in the dark for another 12 h before washing with PBS **(**3 times**)** and incubated with RPMI containing 20** %** of methyl thiazolyl tetrazolium **(**MTT**)** solution** (**5 mg mL**^-^**^1^**)** for another 3 h under the same condition**.** Then, the cells were washed with 3 times PBS before DMSO **(**100 μL**)** was added into each well to dissolve formazan crystal**.** Finally, the absorbance of formazan solutions was detected at 490 nm to determine the relative cell viabilities using a plate reader (Epoch Microplate Spectrophotometer)**.** Furthermore, calcein-AM and PI was co**-**stained to visualize live and dead cells**.** Cells were seeded into 6**-**well plates at 2 x 10^5^ cells**/**well and cultured for 24 h at 37 ºC under 5 **%** CO_2_**.** Pa**-**PEG nanodots were dispersed into RPMI cell**-**culture media with a concentration of 0**.**25 µM**.** After incubation for 8 h, the cells were irradiated by a red LED lamp for 30 min and then incubated for another 24 h**.** After that, 4 µM calcein acetoxymethyl and propidium iodide **(**calcein AM**/**PI, Thermo Fisher Scientific**)** was added to each well and then the cells were incubated for 5 min before imaging by ZOE Fluorescent Cell Imager** (**Bio**-**Rad**)**.

*Cellular uptake***:** 4T1 cells were seeded into 12**-**well plates containing cover glass slips at a density of 1 x 10^5^ cells**/**well and cultured for 24 h**.** Pa**-**PEG nanodots were added into each well at the final concentration of 1 μM of Pa and cultured for various time points **(**1, 3, 8, and 12 h**).** After being washed with PBS** (**3 times**)**, the cells were fixed with 4** %** paraformaldehyde for 10 min and washed with PBS before staining with 4**′**, 6**-**diamidino**-**2**-**phenylindole** (**DAPI**)** for 15 min**.** Later, the cells were imaged by a confocal microscope** (**Leica TCS**-**SP5II, Germany**).** Z**-**stacking images were obtained by a confocal microscope to ensure the accumulation of Pa**-**PEG nanodots inside the cells after 8 h incubation**.** Dose**-**dependent internalization of the nanodots was also determined using flow cytometry referring to the reported procedure 49**.** Concisely, 4T1 cells at a density of 1 x 10^5^ cells treated with Pa**-**PEG nanodots at the final dose of 0, 1**.**25, 2**.**5, 5**.**0, 10 and 20 μM of Pa for 8 h were transferred into Eppendorf tubes and washed with cold PBS**.** The cells were centrifuged at 800 g at 4 °C for 5 min and re-suspended in 500 μL of PBS with 2 times washing**.** After centrifugation, the cells were re-suspended in 500 μL of cold PBS. Then, 20,000 events (cells) were analyzed by flow cytometry using an Attune NxT Acoustic Focusing Cytometer (Life Technologies) using 637 nm for excitation and 730 nm for detecting emission wavelength. Data were acquired and analyzed with Attune NxT software (Life Technologies).

*Intramolecular ROS generation detection***:** the cells were seeded into 12**-**well plates containing cover glass slips at a density of 1 x 10^5^ cells**/**well and cultured for 12 h**.** The cell plates were divided into 4 groups including; **(**i**)** control;** (**ii**)** Pa-PEG only;** (**iii**)** light only; (iv) **Pa-PEG +** light**.** After 8 h incubation, 2**′**,7**′-**dichlorofluoresceindiacetate** (**DCFH**-**DA**) was added** into each well at the concentration of 20 μM**.** Afterward, the cover glass slips of all groups were brought to image using Leica TCS**-**SP5II confocal microscope.

**Tumor models.** 4T1 xenografts were prepared by subcutaneous injection of 1 x 10^6^ cells in PBS** (**~50 μL**)** onto the back of shaved female balb**/**c mice**.** The mice were used when the volume of the tumor reached up to ~150 mm^3^.

***In vivo* Fluorescence Imaging.** Pa**-**PEG nanodots **(**300 μL, 1**.**8 mg mL**^-^**^1^**)** were intravenously injected into 4T1 tumor**-**bearing mice**.** Accumulation of Pa**-**PEG nanodots was monitored using Maestro EX fluorescence imager with 660 nm and 700 nm excitation and emission filters, respectively, and the exposure time of 50 ms**.** After 8 and 24 h post-injection, the mice were sacrificed and the major organs including the liver, spleen, kidney, lung, intestine, stomach, heart and tumor were collected for *ex vivo* distribution**.** Afterward, the tumor and major organs were frozen in optimum cutting temperature **(**OCT**)** solution at **-**80 °C for histology studies.

***In vivo* Photoacoustic Imaging.** The mice were intravenously injected with Pa**-**PEG nanodots and monitored real time accumulation of Pa**-**PEG nanodots at the tumor site and kidney using Visualsonic Vevo 2100 LAZER system with 710 nm excitation**.** Afterward, the mice were sacrificed and the major organs were collected**.** FL and PA signals were displayed as radiant efficiency.

***In vivo* Photodynamic Therapy.** Mice were randomly divided into four groups **(***n* **=** 5**)** for various treatments**: (**i**)** control; **(**ii**)** light only; **(**iii**)** Pa**-**PEG i**.**v**.** injection; and **(**iv**)** Pa**-**PEG i**.**v**.** injection **+** light**.** After 300 μL of Pa**-**PEG nanodots **(**300 μL, 1**.**8 mg mL**^-^**^1^**)** was injected into mice bearing 4T1 tumors, the tumors of group (ii) and (iv) were exposed to a red LED lamp** (**power density** =** 10 mW cm**^-^**^2^**)** for 60 min**.** The tumor sizes were measured every other day using a caliper**.** Tumor volumes were calculated as** (**tumor length**)** ×** (**tumor width**)**^2^**/**2 and *V***/***V_0_*** (***V_0_* is the initial tumor volume**)** was used as relative tumor volumes**.** Two days after treatment, the tumors from each group were embedded in paraffin for histology study.

**Blood Analysis and Histology Examination.** Healthy balb**/**c mice were randomly divided into three groups **(**n **=** 3**)** after intravenously injected of Pa**-**PEG nanodots** (**300 μL, 1**.**8 mg mL**^-^**^1^**).** The mice were sacrificed at the first and seventh day p**.**i**.**, while other three untreated mice were used as the control**.** Subsequent, blood samples **(∼**1 mL for each mouse**)** were collected for blood panel analysis and blood chemistry examination at Cyrus Tang Hematology Center of Soochow University**.** In addition, major organs from each mouse were harvested for histological investigation.

## 3. Results and Discussion

**Figure 1:**
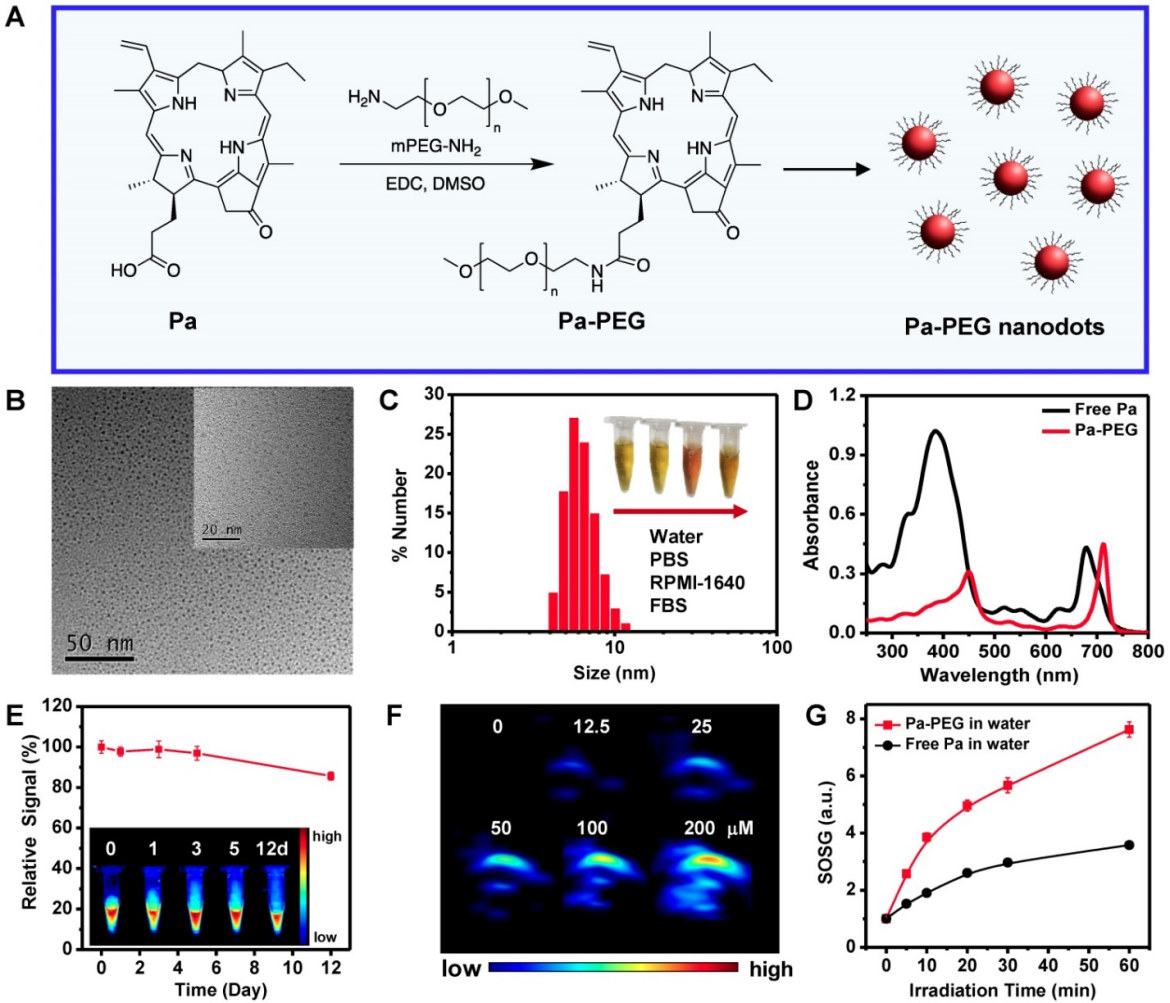
Synthesis, characterization and optical properties of Pa-PEG nanodots. (A) Synthetic scheme and molecular structure of Pa-PEG nanodots. (B) TEM image of Pa-PEG nanodots dispersed in water. Inset: high-magnification TEM. (C) Hydrodynamic diameters of Pa-PEG nanodots after incubation in water. Inset: Photo of Pa-PEG nanodots in various physiological solutions. (D) UV-Vis-NIR spectra of free Pa in DMSO and new characteristic pattern of Pa-PEG nanodots in water. (E) Fluorescence stability of Pa-PEG nanodots over extensive time. Inset: Photograph of fluorescence stability of Pa-PEG nanodots. (F) Photoacoustic images of Pa-PEG nanodots at various concentrations of Pa (0, 12.5, 25, 50, 100, and 200 μM) in water. (G) Singlet oxygen production rates of free Pa and Pa-PEG nanodots in water at the same concentration of Pa (20 ug mL^-1^) were determined by measuring the fluorescence intensity using SOSG as a ^1^O_2_ acceptor upon exposure to the red LED lamp at the power density of 10 mW cm^-2^.

PEGylated pyropheophorbide**-**a was synthesized *via* a general amide coupling condition **(Figure 1A).** In this work, a carboxyl group on pyropheophorbide**-**a **(**Pa**)** was activated with 1**-**ethyl**-**3**-(**3**-(**dimethylamino**)** propyl**)** carbodiimide **(**EDC**)** before reacting with a single terminated NH_2_ polyethylene glycol. Finally, PEGylated**-**Pa** (**Pa**-**PEG**)** was obtained after dialysis against DI water. Pa**-**PEG nanoparticles were dispersed in 9** %** NaCl solution with a ratio volume of 1** :** 1 for stability test and it was found that all Pa**-**PEG could greatly dissolve without any aggregation in such high salt solution**.** In contrast, free Pa showed immediate aggregation in the brine solution due to its less solubility in water** (Figure S1).** Thin layer chromatography **(**TLC**)** displayed no free Pa remained inside the Pa**-**PEG nanoparticles **(Figure S2).** ^1^H nuclear magnetic resonance **(**^1^H**-**NMR**)** showed the characteristic new peaks that belong to PEG around 3**.**5 ppm after PEGylation, suggesting the successful conjugation of PEG on Pa molecule **(Figure S3).** Matrix assisted desorption**/**ionization time**-**of**-**flight **(**MALDI**-**TOF**)** found the molecular mass peak of Pa**-**PEG, which was 5,863 m**/**z** (Figure S4).** Transmission electron microscopy **(**TEM**)** image revealed the obtained nanoparticles were uniform nanodots morphology with 2**.**28 ± 0**.**45 nm in size** (Figure 1B, Figure S5).** The hydrodynamic **(**HD**)** size was measured to be 5.76 ± 0.56 nm **(Figure 1C)**, a little larger than the size from TEM, this might be due to the HDs that corresponds to the core and the swollen corona of nanoparticles 35, 50, 51**.** In addition, red**-**shift of Soret and Q bands in absorption spectra after modification suggested that Pa molecule in adducts might be contributed to form nanodot morphology *via* π**-**π stacking** (Figure 1D).** Moreover, the periphery of hydrophilic PEG moieties was able to stabilize the nanodots structure in water during the self**-**assembly process to maintain individual nanodot 52-54**.** The zeta**-**potentials of Pa**-**PEG nanodots and free Pa in DI water were **-**5**.**70 ± 1**.**37 and **-**11**.**76 ± 1**.**46 mV, respectively, which prone to have renal eliminable properties** (Figure S6).** Moreover, Pa**-**PEG nanodots have also demonstrated long**-**time solubility and stability in DI water and FBS solution for at least 15 days** (Figure S7)**.

UV**-**Vis**-**NIR spectra exhibited the red**-**shift of absorption spectra in H_2_O after PEGylation with remarkable absorption maxima shifted with increasing sharpness from 384 to 450 nm and 680 nm to 710 nm** (Figure 1D).** To ensure the optical properties of Pa**-**PEG nanodots serving as imaging**-**guided PDT, fluorescent emission and photoacoustic effect were investigated**.** Upon excitation, Pa**-**PEG nanodots displayed a strong emission peak at 710 nm, denoting the good property for *in vivo* fluorescence imaging**.** In contrast, free Pa was mostly quenched in aqueous solution**.** The standard curve between average FL signal at 660 nm excitation against various concentrations ranging from **3-28** μM was linear increasing with R^2^** =** 0**.**9870** (Figure S8).** In addition, noticeable photostability was observed under 1000 ms of exposure time at 660 nm excitation as the Pa**-**PEG nanodots** (**30 μM**)** could maintain good fluorescence at least 12 days with trivial photodegradation**.** Moreover, the photograph of FL imaging of Pa**-**PEG nanodots also exhibited strong fluorescent signals up to 12 days** (Figure 1E).** The PA optical property was evaluated using various concentrations of Pa**-**PEG nanodots ranging from 0**-**200 μM and it was found that the nanodots could produce high PA signal upon excitation at 710 nm and the intensity was dose**-**dependent manner showing as a linear correlation with R^2^** =** 0**.**9866** (Figure 1F, Figure S9).** Singlet oxygen quantum yield **(**Ф**_∆_)** of free Pa and Pa**-**PEG nanodots were quantified using DPBF as a singlet oxygen quencher**.** The absorbance of DPBF photoconsumption during a red LED lamp** (**600~700 nm**)** irradiation were recorded by keeping track of the DPBF maximum absorption at 418 nm** (Figure S10A).** Based on first**-**order plots in **Figure S10B**, the Ф**_∆_** of free Pa nanodots was 55**.**7** %** regarded to zinc phthalocyanine as the reference compound **(**Ф**_∆_ =** 67 **%** in DMSO**)** 48**.** Simultaneously, the Ф**_∆_** of free Pa**-**PEG nanodots was acquired as 53**.**6** %**, which was no significant difference compared to that of free Pa**.** Furthermore, singlet oxygen production was also monitored by detecting the fluorescent intensity of singlet oxygen sensor green **(**SOSG**)** at 494 nm after a red LED lamp irradiation of Pa**-**PEG nanodots**.** Under the same Pa concentration, the fluorescent signals of SOSG obtained from irradiated Pa**-**PEG nanodots were higher compared to those from free Pa molecule, indicating that more ^1^O_2_ could be generated which might be due to the better solubility of Pa in water after PEGylation **(Figure 1G).** These optical properties results suggest that our Pa**-**PEG nanodots might serve as a powerful agent for PA**/**FL imaging**-**guided PDT.

**Figure 2:**
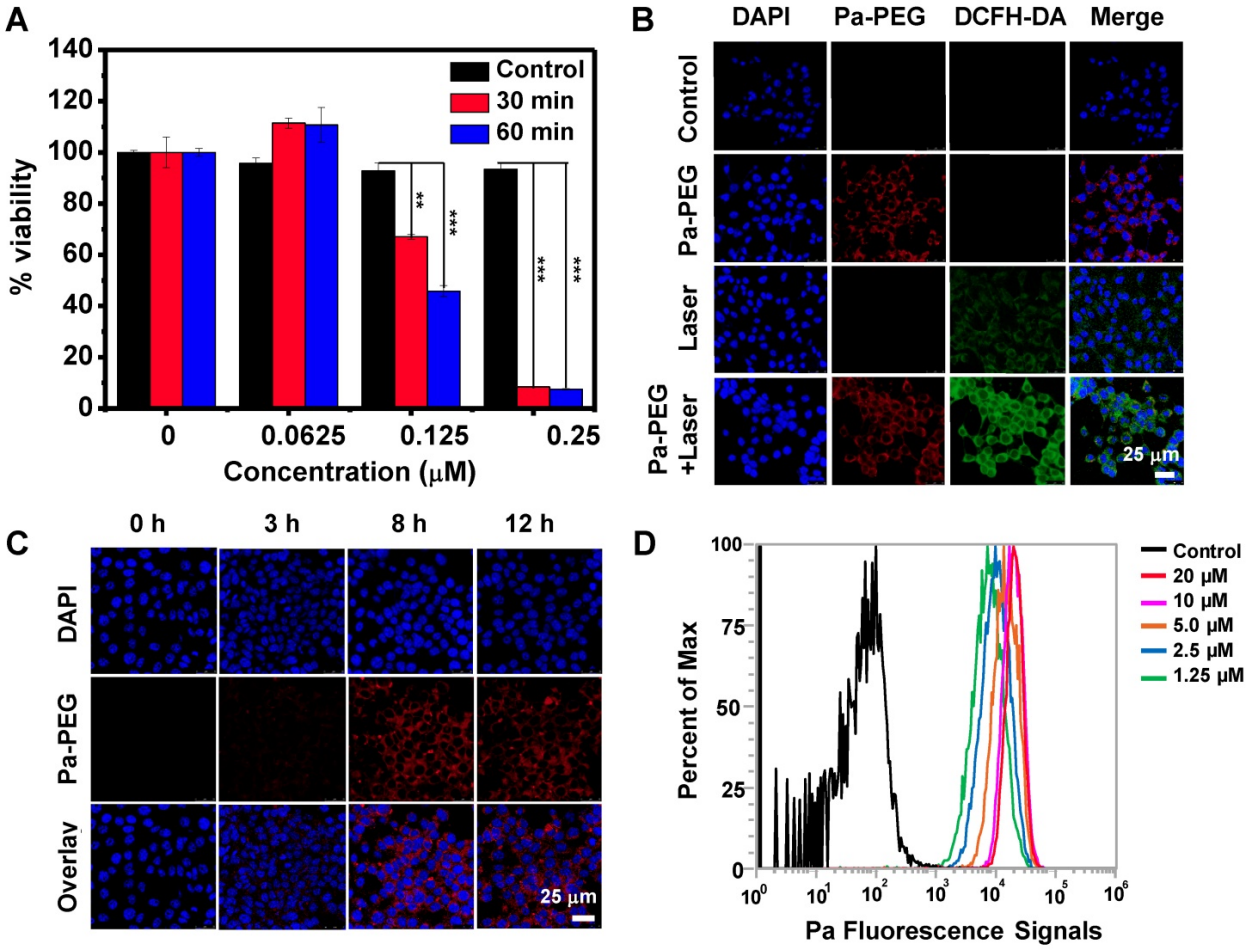
*In vitro* studies of Pa-PEG nanodots. (A) Relative cells viabilities of 4T1 cells treated Pa-PEG nanodots at different Pa concentrations for 8 h and irradiated with a red LED lamp at power density of 10 mW cm^-2^ for 30- and 60-min. Statistical analysis are based on independent t-test (***p < 0.001, **p < 0.01, or *p < 0. 05). (B) *In vitro* ROS production of various treatments in 4T1 cells using DCFH-DA as a singlet oxygen acceptor. Scale bars = 25 μm. (C) Cellular uptake of Pa-PEG nanodots in 4T1 cells at 0, 3, 8, and 12 h. (D) The histogram presents the increasing of fluorescence signals in 4T1 cells as a function of concentrations at 0, 1.25, 2.5, 5.0, 10, and 20 μM.

*In vitro* cytotoxicity of Pa**-**PEG nanodots was tested in 4T1 murine breast cancer cells using the standard methyl thiazolyl tetrazolium **(**MTT**)** assay**.** The cells maintained full viability when they were treated with Pa**-**PEG nanodots up to 20 μM for 24 h *without* irradiation** (Figure S11).** On the other hand, the cells incubated with 0**.**25 μM of Pa**-**PEG nanodots and exposed to a red LED lamp for 30 min were mostly destroyed** (Figure 2A).** Next, calcein**-**AM and propidium iodide **(**PI**)** co**-**staining was also performed to confirm Pa**-**PEG nanodots triggered by a red light could induce cell death while the non**-**radiated cells remained alive **(Figure S12). In addition, t**o ensure the presence of reactive oxygen species **(**ROS**)** inside the cells after irradiation, dichloro**-**dihydro**-**fluorescein diacetate **(**DCFH**-**DA**)** assay was conducted to visualize the existing of ROS**.** Non**-**fluorescence** 2**',**7**'**-**dichlorodihydrofluorescein diacetate was oxidized by ROS**-**mediated oxidation to obtain the green fluorescence of 2',7'**-**dichlorofluorescein **(**DCF**)** 46**.** As shown in** Figure 2B**, bright green fluorescence from DCF was significantly enhanced when the cells treated with Pa**-**PEG nanodots were irradiated compared to the other control groups**.** Cellular uptake was also performed at different time points 0, 3, 8, and 12 h and monitored by a confocal microscope** (Figure 2C). The r**ed fluorescent signal was observed from 4T1 cells treated with Pa**-**PEG nanodots after incubation for 3 h and the brightness increased to a maximum after 8 h incubation**.** In contrast, according to the quenched fluorescent signal of free Pa in water, the red fluorescent signal of free Pa was barely observed inside the cells** (Figure S13A).** Furthermore, the internalization of Pa**-**PEG nanodots was quantified by flow cytometry (**Figure 2D**), confirming that the Pa**-**PEG nanodots were localized inside the cells in a does**-**dependence manner**.** Moreover, the cellular uptake of Pa**-**PEG nanodots was also visualized by z**-**stacking confocal images** (Figure S13B).** All these results suggested that Pa**-**PEG nanodots could effectively destroy the cancer cells under a red LED lamp light**-**triggered PDT effect.

**Figure 3:**
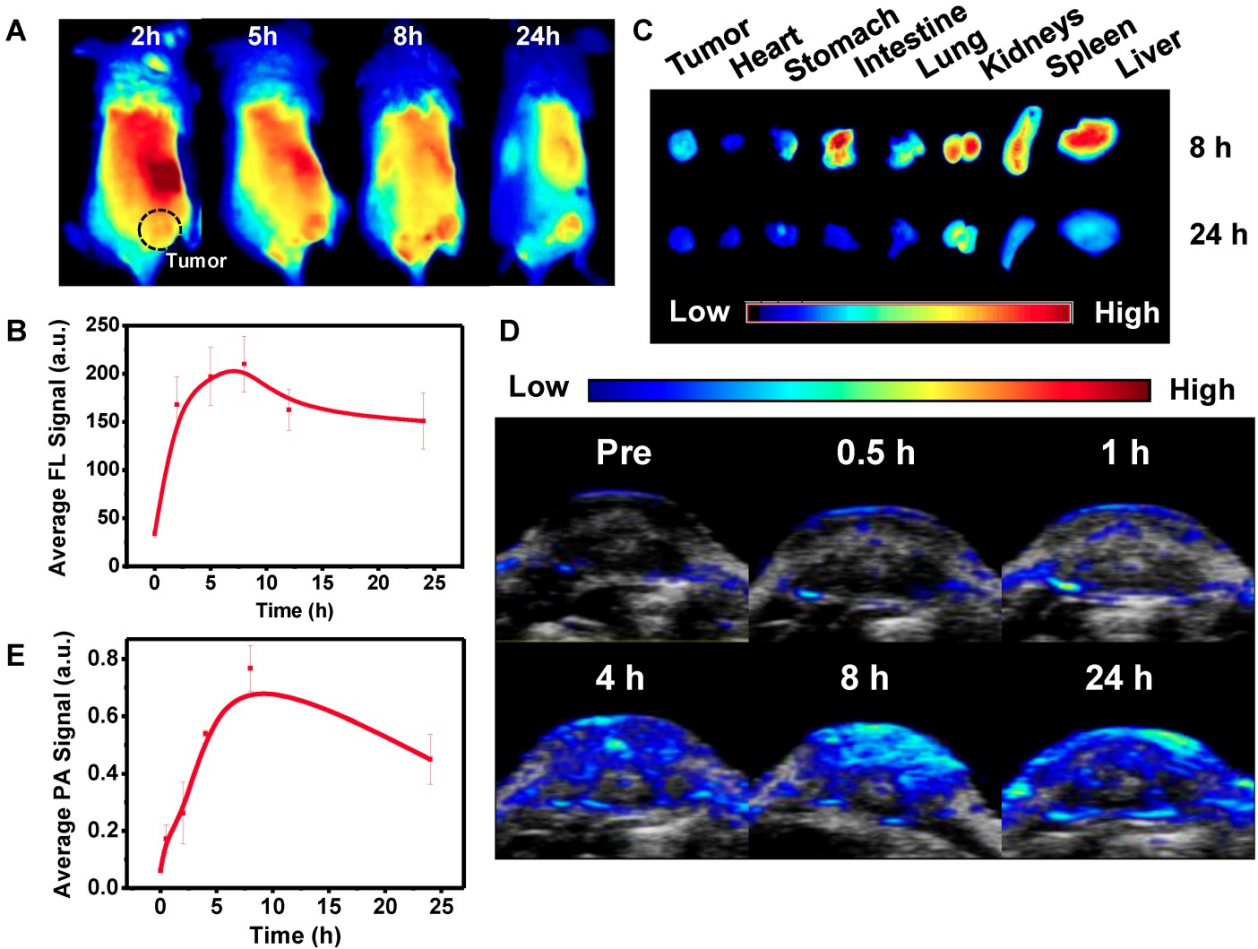
*In vivo* fluorescence and photoacoustic imaging of Pa-PEG nanodots. (A) Fluorescence images of 4T1 tumor bearing mice post injecting Pa-PEG nanodots (1.8 mg mL^-1^, 300 μL) at different time points. (B) Fluorescence signal analysis of the tumor at different time points p.i. (C) *Ex vivo* fluorescence images of main organs and tumor were obtained 8 and 24 h p.i. (D) Photoacoustic images of tumor post injecting Pa-PEG nanodots (1.8 mg mL^-1^, 300 μL) at different time points. (E) Photoacoustic intensities of localized Pa-PEG nanodots at tumor site with the time increased.

Optimal time of Pa**-**PEG nanodots accumulation at the tumor site was monitored by *in vivo* PA**/**FL dual**-**modal imaging**.** First, Pa**-**PEG nanodots **(**300 μL, 1**.**8 mg mL**^-^**^1^ Pa concentration**)** were intravenously injected into 4T1 tumor**-**bearing mice and then the fluorescent signal was monitored at various time points**. Figure 3A** showed that Pa**-**PEG nanodots circulated rapidly through the whole body and were accumulated at the tumor site since the first 2 h post injection **(**p**.**i**.)**, and then the accumulation continuously increased to reach the maximum at 8 h p**.**i**. (Figure 3B).** Major organs including liver, spleen, kidney, lung, intestine, stomach, heart, and tumor were taken out at 8 and 24 h p**.**i**.** for *ex vivo* fluorescence imaging and the quantitative fluorescent biodistribution was analyzed **(Figure 3C, Figure S14).** Strong fluorescent signals were observed from the organs at 8 h including liver, spleen, kidney, and intestine. The moderate signals were observed from tumor due to the equilibrium between tumor**-**targeting ability and clearance ability of Pa**-**PEG nanodots. The high signal at stomach was from the food background**.** At 24 h p**.**i**.**, most of the nanodots were cleared from the major organs including tumor. However, the signal from kidney was still clearly observed due to long blood circulation of the nanodots that the size was small enough to move through the glomerular cell membrane to be excreted out of the body *via* the urinary system 55**.** After intravenous injection of Pa-PEG nanodots for half an hour, the high fluorescent signal was found in the urine, which indicated that the ultrasmall Pa-PEG nanodots were cleared by renal removing pathway (**Figure S15**). After 8 h p**.**i**.**, major organs were sectioned and imaged under confocal microscopy to confirm the uptake of Pa**-**PEG nanodots**.** Strong red fluorescence was observed in liver, spleen, kidney, and intestine, and the moderate signal was observed from the tumor, indicating the existence of Pa**-**PEG nanodots in those organs** (Figure S16).**

Next, *in vivo* PA imaging was conducted to ensure that Pa**-**PEG nanodots could serve as dual**-**modal imaging agent for cancer therapy, and to confirm the retention of Pa**-**PEG nanodots at the tumor site** (Figure 3D-E).** Pre**-**injection was firstly imaged, and then time**-**dependent PA imaging was continued after i**.**v**.** injection of Pa**-**PEG nanodots **(**300 μL, 1**.**8 mg mL**^-^**^1^**)** into 4T1 tumor**-**bearing mice**.** PA signal at tumor region showed tumor uptake over time and the nanodots could obviously be retained to the highest distribution at 8 h p**.**i**.** before gradually decreased as the function of time until 24 h p**.**i**.**, which was similar to the FL imaging results**.** Therefore, Pa**-**PEG nanodots could be an excellent guiding imaging agent for *in vivo* cancer therapy.

**Figure 4:**
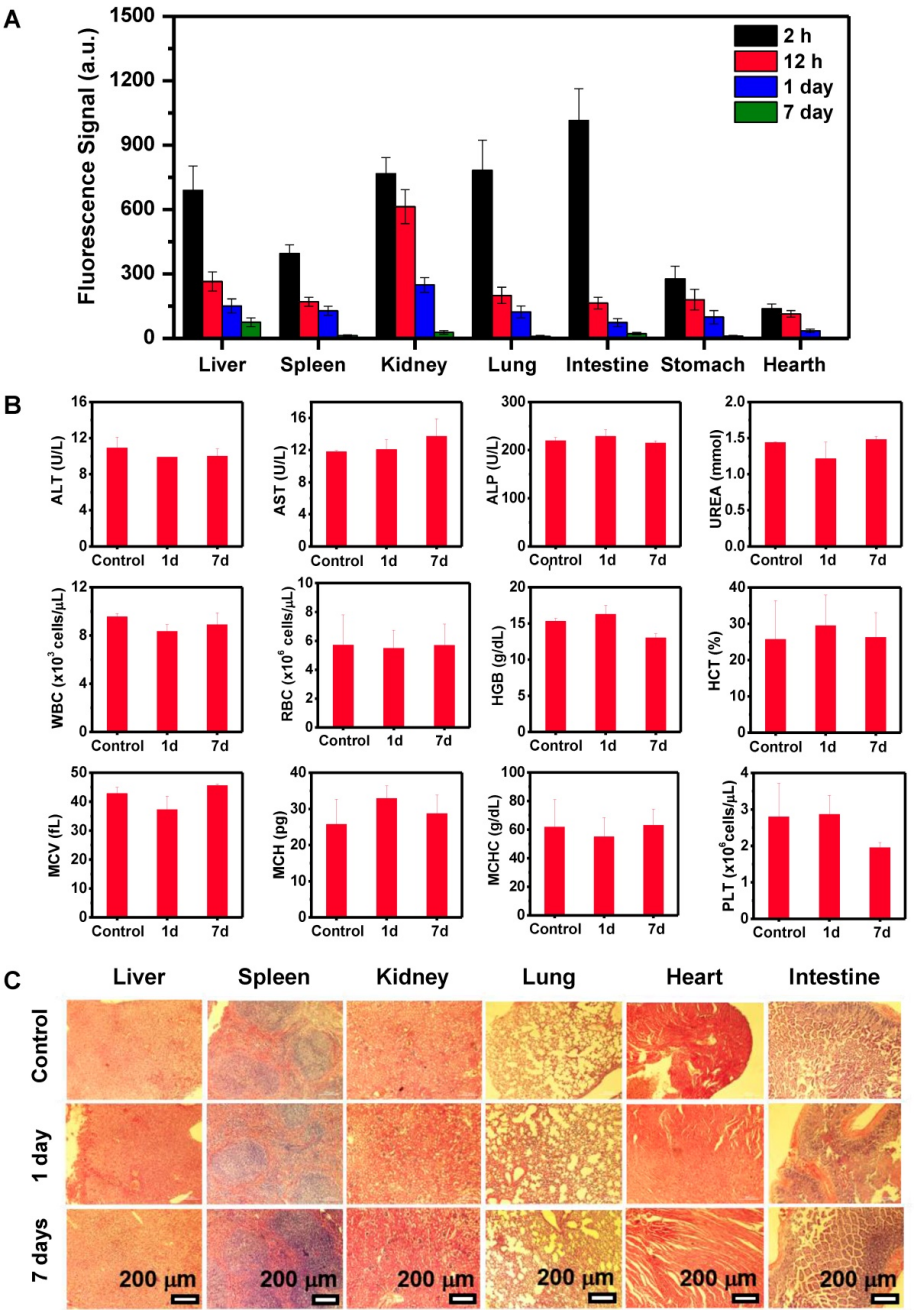
Clearance behavior and toxicity of Pa-PEG nanodots. (A) Biodistribution of Pa-PEG nanodots in major organs showed clearable behavior over time increased. (B) Blood routine and blood biochemistry analysis of untreated female balb/c mice (control) and intravenous administration of Pa-PEG nanodots (1.8 μg mL^-1^, 300 μL), which their bloods were collected at 1- and 7-day p.i. (n = 3). (C) H&E stained major organs include liver, spleen, kidney, lung, heart, and intestine at different time points of untreated mice, 1 day, and 7 days p.i.

In general, nanoparticles containing size less than 8 nm and negative charge on the surface can be an advantage for renal clearable 35, 36**.** Thus, Pa**-**PEG nanodots with about 5.76 nm** (**HD**)** in size and negative zeta potential were also investigated for renal clearance behavior**.** Time**-**dependent PA imaging was applied to visualize PA signal in mouse kidneys and the results showed that the signal was risen up during the first 2 h, suggesting the filtration at kidney was allowed by blood circulation after injecting Pa**-**PEG nanodots** (Figure S17).** After 2 h p**.**i**.**, the organs were taken out for *ex vivo* PA imaging and the results exhibited Pa**-**PEG nanodots were observed in kidneys as well as other organs **(Figure S18). The** PA biodistribution pointed out the major accumulation of Pa**-**PEG nanodots was in the liver, spleen, and kidneys, suggesting that the blood circulation could provide physical filtration of Pa**-**PEG nanodots at kidney implying the renal pathway activation, while the accumulation in other organs was reabsorbed and circulated for further elimination 56**.** Similar experiments were conducted using FL imaging** (Figure S19).** *Ex vivo* FL images of major organs at various time points showed time**-**dependent biodistribution with strong Pa**-**PEG nanodots signal at the first 2 h p**.**i**.** in liver, spleen, kidney, lung, and intestine**.** The uptake of liver, spleen, lung, and intestine was promptly reduced within 12 h whereas the FL signal from the kidneys remained at a high level compared to other organs, suggesting that Pa**-**PEG nanodots could be cleared from the body through the kidneys *via* the urinary system**.** Relative FL intensities at 7**-**day p**.**i**.** revealed the disappearance of signals in several organs including spleen, kidney, lung, intestine, and heart because of the clearance compared with that at 2 h p**.**i**. (Figure 4A).** Pa**-**PEG nanodots remained in the liver might enter the bile *via* the hepatic circulation system, and excreted in feces 56**.** Notably, it was found that at 2 h p.i. PA signal in the liver was slightly higher than that of the kidney, while FL signals in the liver and kidneys were about the same intensities**.** These might be because a large amount of Pa**-**PEG nanodots in the liver and intestine from both imaging techniques came from a cooperative excretion *via* bile clearance with an entero**-**hepatic cycle that cause biodistribution variation in the liver and intestine at the first 2 h, and *via* renal elimination 25**.** However, the renal removing pathway is dominant clearable behavior for long**-**term drainage owning to their ultra-small size.

Blood routine and blood biochemistry were examined to evaluate *in vivo* long**-**term toxicity of Pa**-**PEG nanodots** (Figure 4B).** In blood routine examination, several variables were investigated, including white blood cells **(**WBC**)**, red blood cells **(**RBC**)**, hemoglobin **(**HGB**)**, hematocrit **(**HCT**)**, mean corpuscular volume **(**MCV**)**, mean corpuscular hemoglobin concentration **(**MCHC**)**, and platelet **(**PLT**).** In addition, the investigated parameters of blood biochemistry were Aspartate aminotransferase **(**AST**)**, Alanine aminotransferase **(**ALT**)**, Alkaline phosphatase **(**ALP**)**, and UREA**.** It was found that ALT slightly dropped after 1**-**day p**.**i**.**, while there was no significant variant in AST, ALT, and UREA level indicating that the function of liver and kidney were normal 46**.** Consequently, all of fluctuating parameters of blood routine and blood biochemistry at 1**-**day could be recovered to the similar amount as the untreated group within 7**-**day p**.**i**.** Furthermore, H&E staining of main organs demonstrated no noticeable eradication of tissues for long**-**time treatment **(Figure 4C).** These all results suggested that the inflammatory disorder response was rapidly improved through the rapid clearance of Pa**-**PEG nanodots over 7 days without tissue destruction, therefore, the Pa**-**PEG nanodots could be safely used for *in vivo* long**-**term imaging**-**guided PDT.

**Figure 5:**
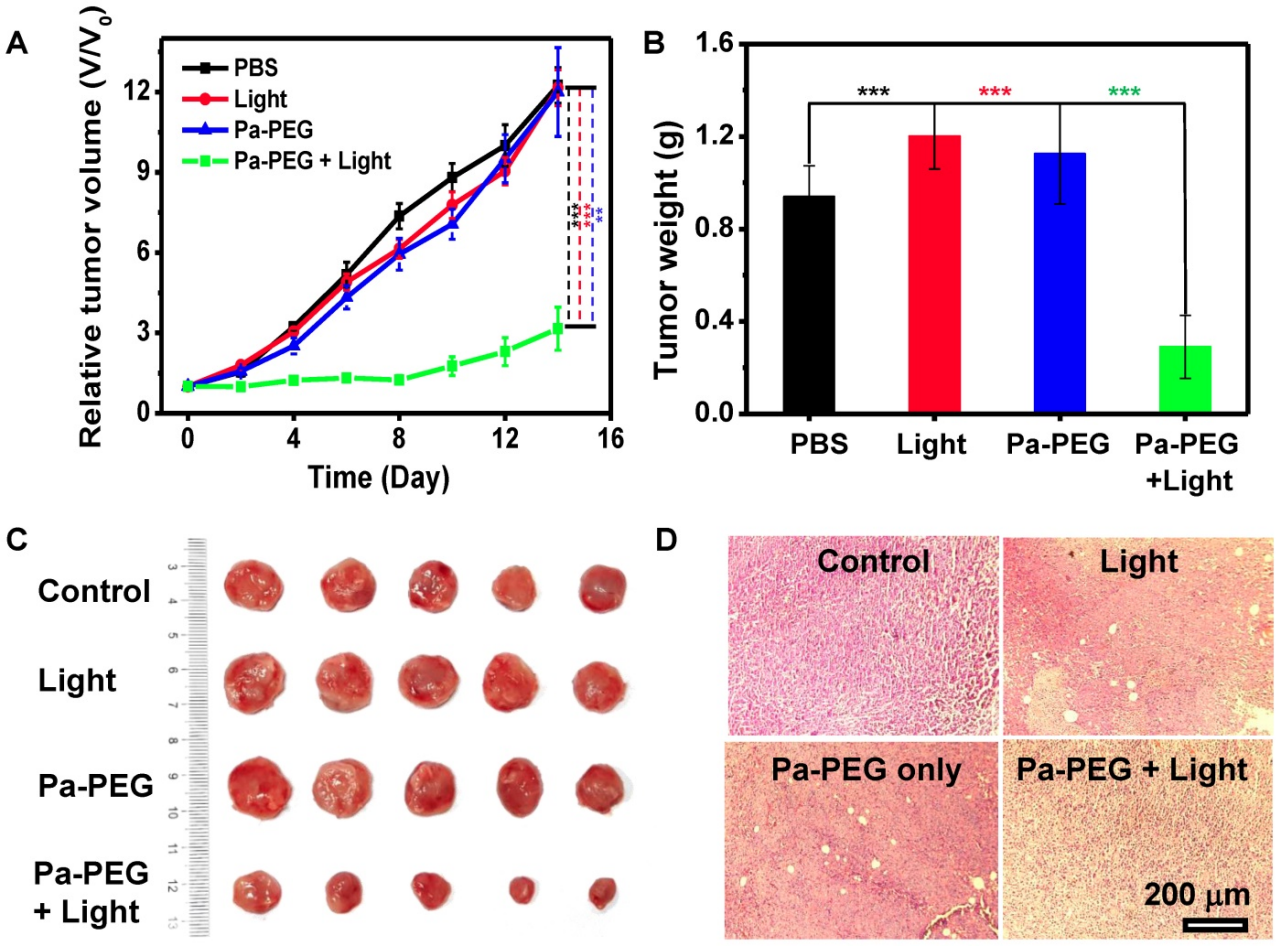
*In vivo PDT*. (A) The tumor growth curves of different 4 groups (n = 5) of Balb/c mice after various treatment. For the treatment group, the mice were received Pa-PEG nanodots and exposed to a red LED lamp (10 mW cm^-2^ for 1 h) at 8 h p.i. For other three groups, the mice were used as controls: untreated (control); light only without Pa-PEG nanodots; Pa-PEG injected without light irradiation. Statistical analysis are based on independent t-test (***p < 0.001, **p < 0.01, or *p < 0.05). (B) Tumor weight of different groups taken at the 14 days. (C) Photographs of the tumor tissue at the 14 days. (D) H&E staining of tumor after various treatments.

Finally, *in vivo* PDT was investigated using 4T1 xenograft mice**.** Twenty mice were randomly divided into 4 groups** (**n **=** 5**)**;** (**i**)** control with no injection, **(**ii**)** a red LED lamp irradiation only **(**10 mW cm**^-^**^2^, 60 min**)**,** (**iii**)** Pa**-**PEG nanodots only** (**300 μL, 1**.**8 mg mL**^-^**^1^**)**, and** (**iv**)** Pa**-**PEG nanodots with a red LED lamp irradiation at 8 h p**.**i**..** Tumor size was measured every other day for 14 days using a caliper**. Figure 5A** shows that tumors from the treatment group were significantly affected by the eradication from photodynamic phenomena after light exposure, while tumors from other three control groups were continuously grown in a similar manner over the period of 14 days**.** Tumor weight and size at day 14 also confirmed the effectiveness of light**-**induced PDT with Pa**-**PEG nanodots treatment **(Figure 5B-C).** The comparison between the representative mouse from each group at day 0 and day 14 clearly established Pa**-**PEG nanodots as a powerful PDT agent** (Figure S20).** Moreover, H&E staining conducted after 16 h post**-**treatment showed that most of the cancerous cells were destroyed, whereas the other three non**-**treatment groups exhibited no significant transformation of cell morphology compared with the control group** (Figure 5D).** Furthermore, the body weight rate of all groups was monitored throughout the treatment period and no significant weight loss was observed** (Figure S21)**.

## 4. Conclusion

In conclusion, ultra**-**small Pa**-**PEG nanodots were successfully synthesized using a simple amide bond formation reaction between PEG and Pa**.** Optical properties demonstrated that the Pa**-**PEG nanodots could be dual**-**modal PA**/**FL imaging agents to track their accumulation at the tumor site with great renal disposing behavior, which accompanied with the ability to serve as an excellent therapeutic nanoagent for PDT**.** *In vitro* studies confirmed the internalization of the nanodots and the production of ROS that could induce cell deaths upon excitation**.** Moreover, Pa**-**PEG nanodots could inhibit tumor growth after PDT treatment guided by PA**/**FL imaging**.** Most of the nanodots were rapidly eliminated from the body *via* renal excretion due to their ultra**-**small diameter**.** In addition, no long**-**term toxicity of the nanodots was observed in our study**.** Therefore, the porphyrin**-**based nanomaterials are promising tumor indicator and can serve as a powerful nanoagent in clinical application for photodynamic cancer therapy.

## Supplementary Material

Supplementary characterization results of Pa**-**PEG nanodots; the stability of nanodots in the salt solution, TLC picture, ^1^H**-**NMR, MALDI**-**TOF, diameter distribution from TEM, zeta**-**potentials, DLS measurements for stability test, fluorescent spectra and linear relationship of FL and PA signal**.** UV-Vis absorption of DPBF photodecomposition at 418 nm, first-order plots, relative cells viabilities in dark condition, fluorescence imaging of calcein-AM/PI co-stained 4T1 cells, confocal imaging of cellular uptake of free Pa, z-stacking imaging, FL quantitative biodistribution, fluorescent image of collected urine, confocal imaged tissue slides, real time PA signal investigation, *ex vivo* PA quantitative biodistribution, *ex vivo* fluorescent images, photographs of mice, body weight curves.
