# Supplementary material for: Ultra-small Pyropheophorbide-a Nanodots for Near-infrared Fluorescence/Photoacoustic Imaging-guided Photodynamic Therapy
Source: Theranostics. 2020 Jan 1;10(1):62–73. doi: 10.7150/thno.35735 (PMC6929619; doi:10.7150/thno.35735)
Supplement: Supplementary file 1 — Supplementary characterization results of Pa-PEG nanodots; the stability of nanodots in the salt solution, TLC picture, 1H-NMR, MALDI-TOF, diameter distribution from TEM, zeta-potentials, DLS measurements for stability test, fluorescent spectra and linear relationship of FL and PA signal. UV-Vis absorption of DPBF photodecomposition at 418 nm, first-order plots, relative cells viabilities in dark condition, fluorescence imaging of calcein-AM/PI co-stained 4T1 cells, confocal imaging of cellular uptake of free Pa, z-stacking imaging, FL quantitative biodistribution, fluorescent image of collected urine, confocal imaged tissue slides, real time PA signal investigation, ex vivo PA quantitative biodistribution, ex vivo fluorescent images, photographs of mice, body weight curves. [file thnov10p0062s1.pdf]

Supporting Information

Ultra-small Pyropheophorbide-a Nanodots for Near-infrared Fluorescence/ Photoacoustic Imaging-guided Photodynamic Therapy

Kittipan Siwawannapong[1], Rui Zhang[2], Huali Lei[2], Qiutong Jin[2], Wantao Tang[2], Ziliang Dong[2], Rung-Yi Lai[1], Zhuang Liu[1], Anyanee Kamkaew[1]*, and Liang Cheng[2]*

1. School of Chemistry, Institute of Science and Center of Excellent-Advanced Functional Materials, Suranaree University of Technology, Nakhon Ratchasima 30000, Thailand.
2. Institute of Functional Nano & Soft Materials (FUNSOM), Collaborative Innovation Center of Suzhou Nano Science and Technology, Soochow University, Suzhou, Jiangsu 215123, China.

Email: lcheng2@suda.edu.cn; anyanee@sut.ac.th

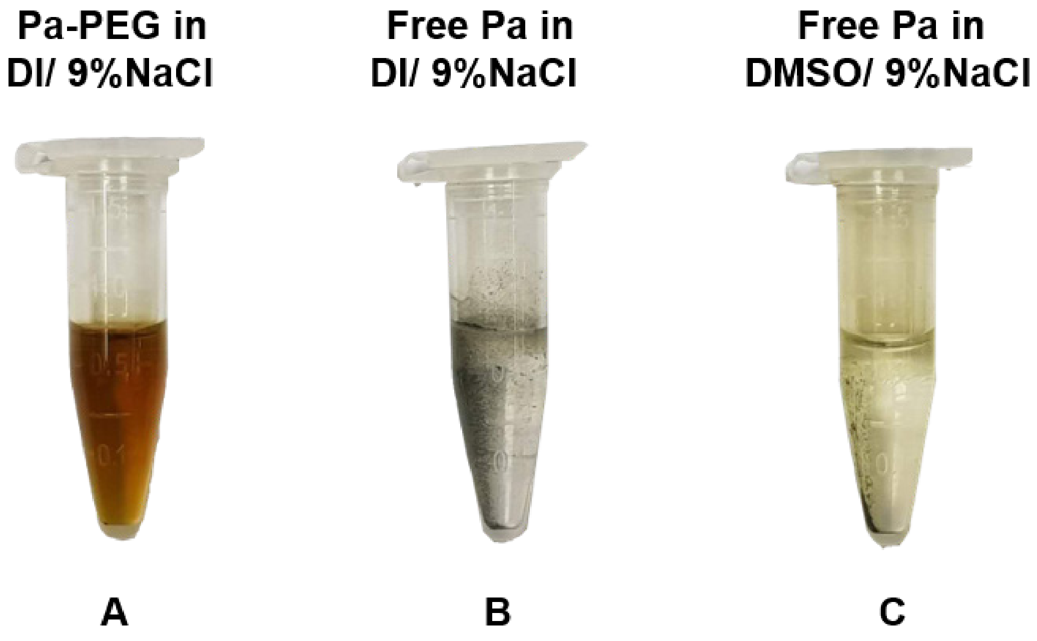

Figure S1. The stability observation after centrifugation of Pa-PEG nanodots and free Pa in salt solution. (A) Most of Pa-PEG nanodots are stable in 9% NaCl salt solution without any aggregation (B) Free Pa molecules have naturally less solubility in DI water, no change was observed after 9%NaCl adding. (C) Free Pa have great solubility in DMSO whereas it instantaneously showed aggregation after the introduction of 9% NaCl.

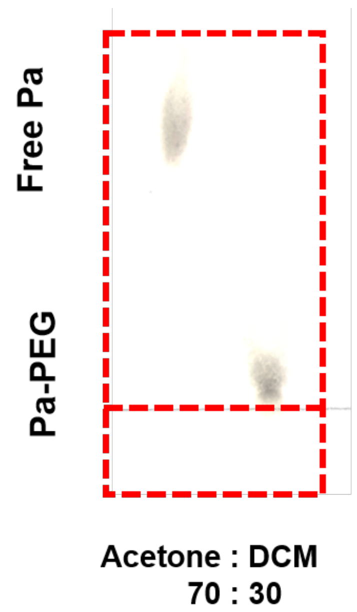

Figure S2. Thin layer chromatography was carried out using acetone: dichloromethane (DCM) with the ratio of 7 : 3 as mobile phase and silica as stationary phase.

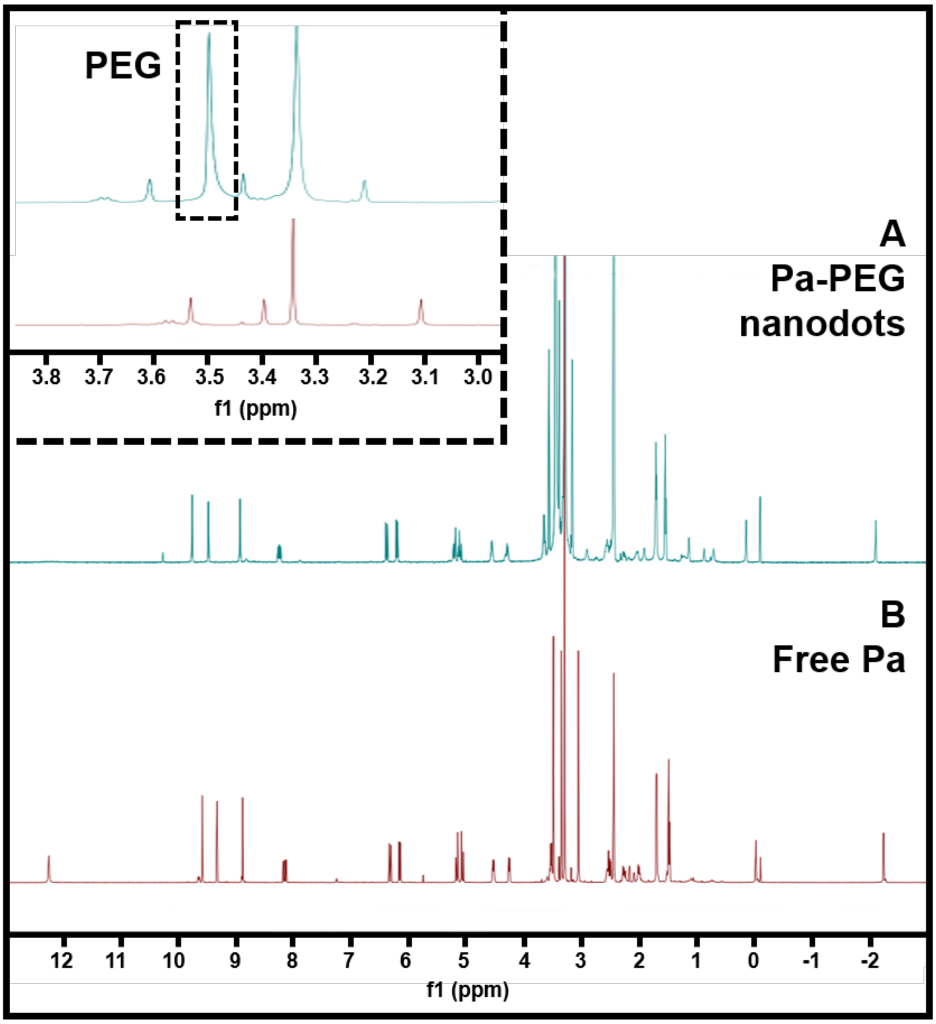

Figure S3. ^{1}H-NMR spectra of (A) Pa-PEG nanodots, (B) free Pa in DMSO. Inserted figure confirmed the existence of PEG after PEGylation.

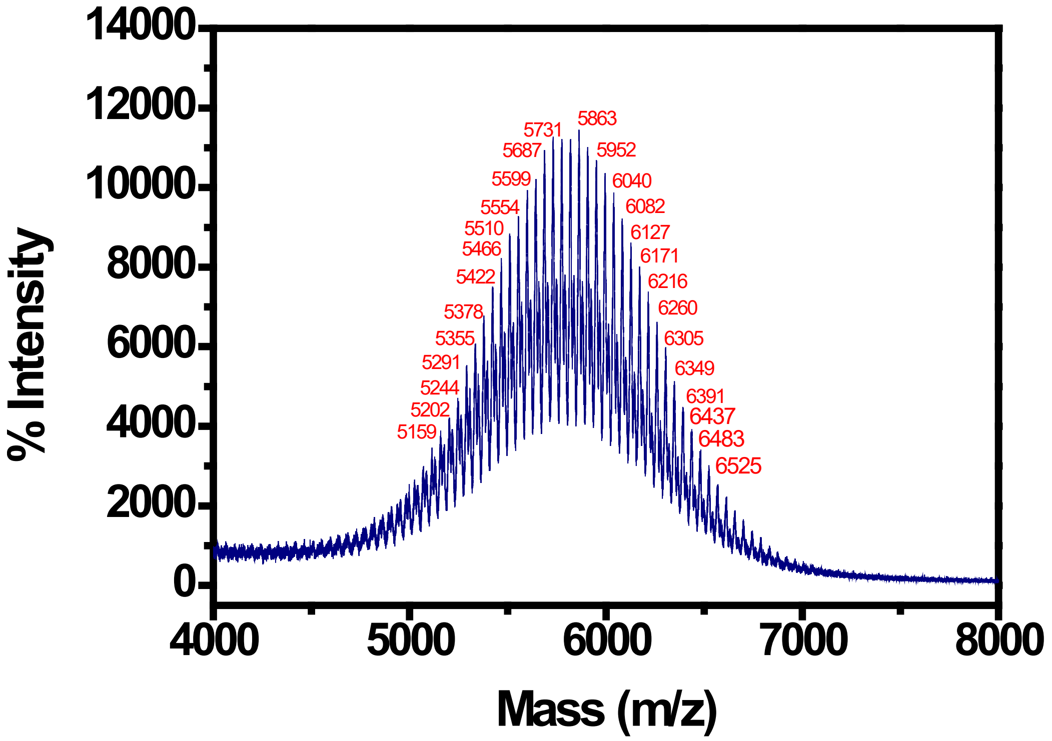

Figure S4. Molecular mass of the Pa-PEG nanodots was determined by matrix-assisted desorption/ionization time-of-flight (MALDI-TOF) mass spectrometry.

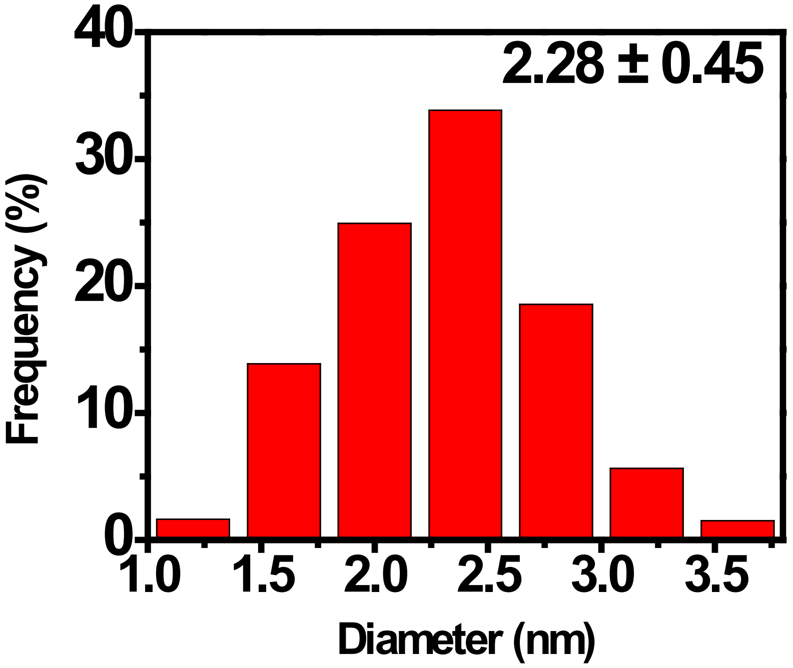

Figure S5. The diameter measurement of Pa-PEG nanodots.

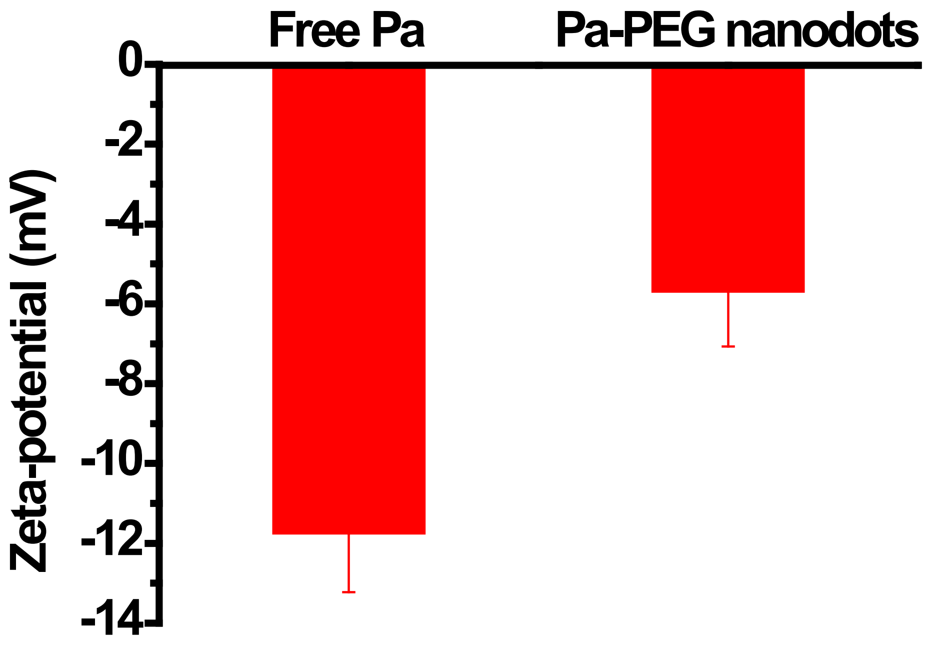

Figure S6. The zeta potential of free Pa and Pa-PEG nanodots were measured by dynamic light scattering (DLS). Error bars were from three separated experiments.

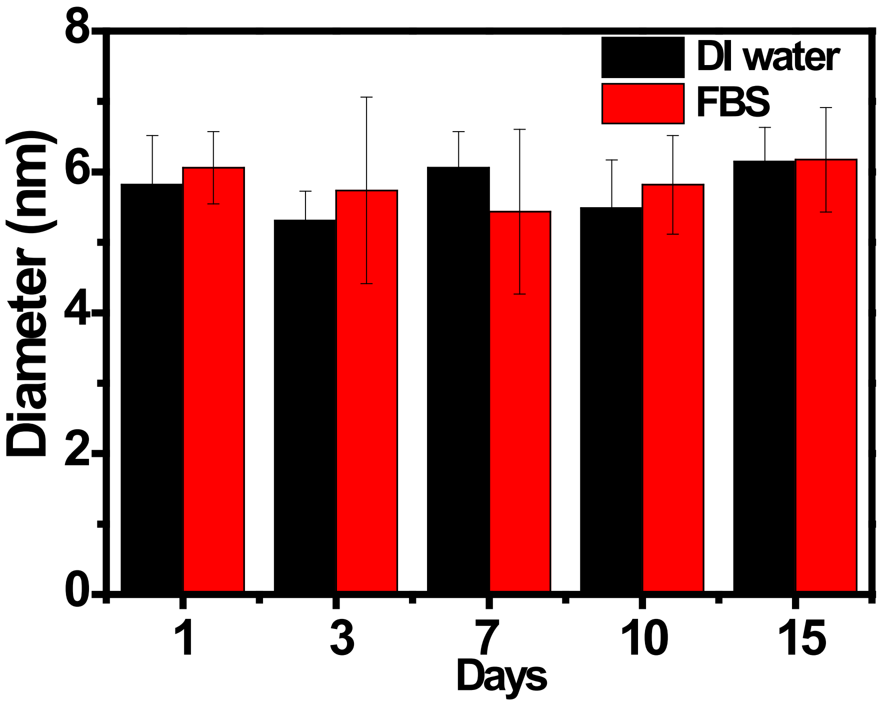

Figure S7. DLS size measurements of Pa-PEG nanodots after being incubated in DI water and FBS for 15 days

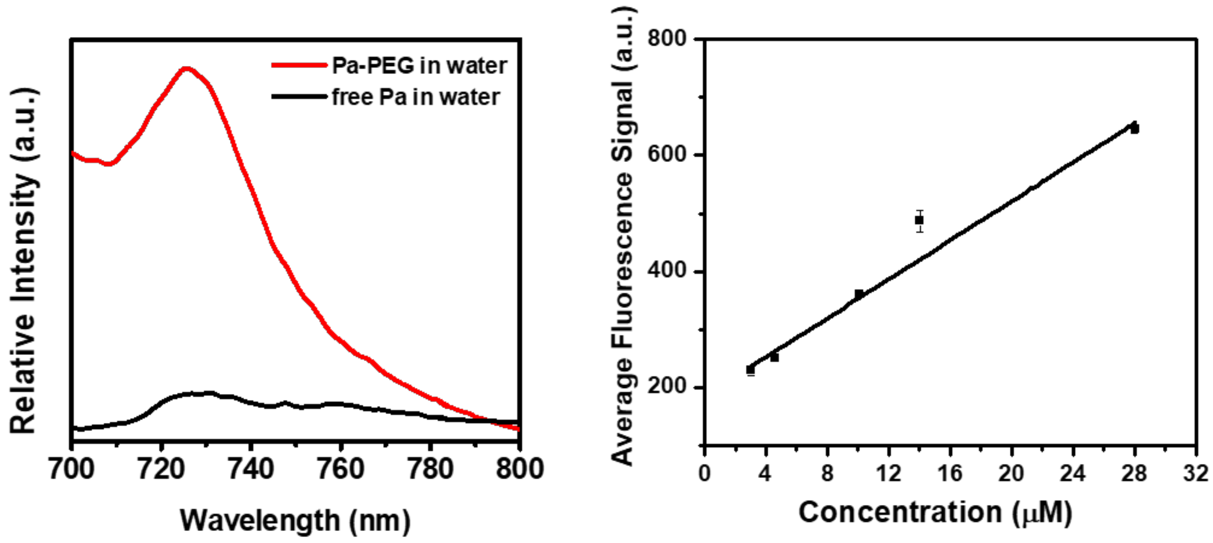

Figure S8. (A) The fluorescent spectra of free Pa and Pa-PEG nanodots in water. (B) The linear relationship of fluorescence signal of Pa-PEG nanodots.

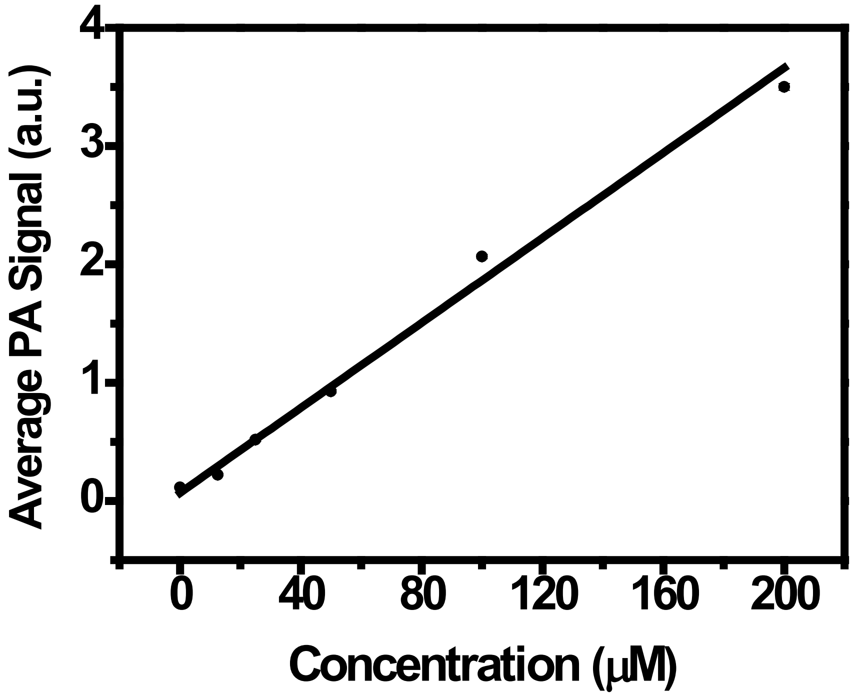

Figure S9. The linearity curve of photoacoustic signal against various concentrations.

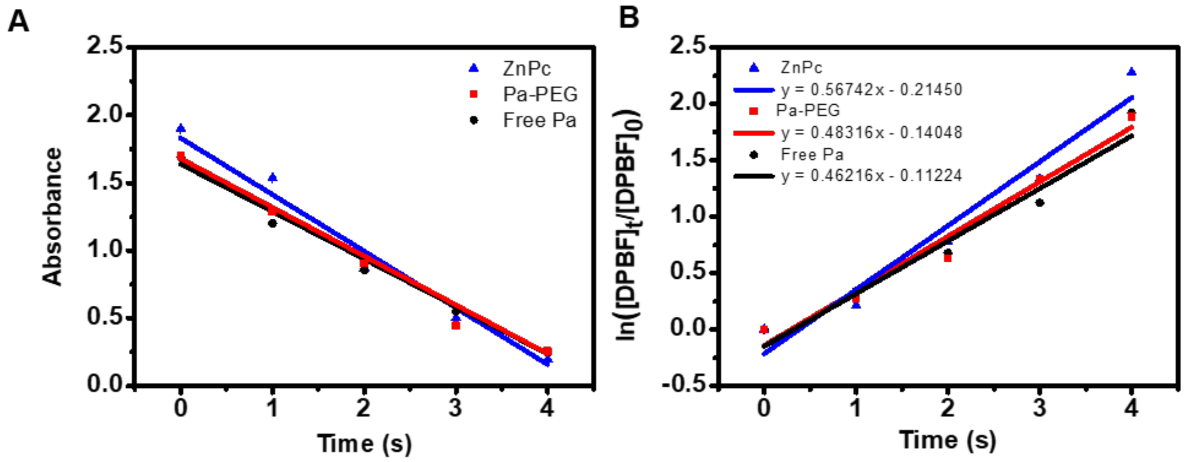

Figure S10. UV-Vis absorption (A) of DPBF photodecomposition at 418 nm during reacting with $^1O_2$ under a red LED lamp irradiation. (B) The first-order plots of photo consumption rate.

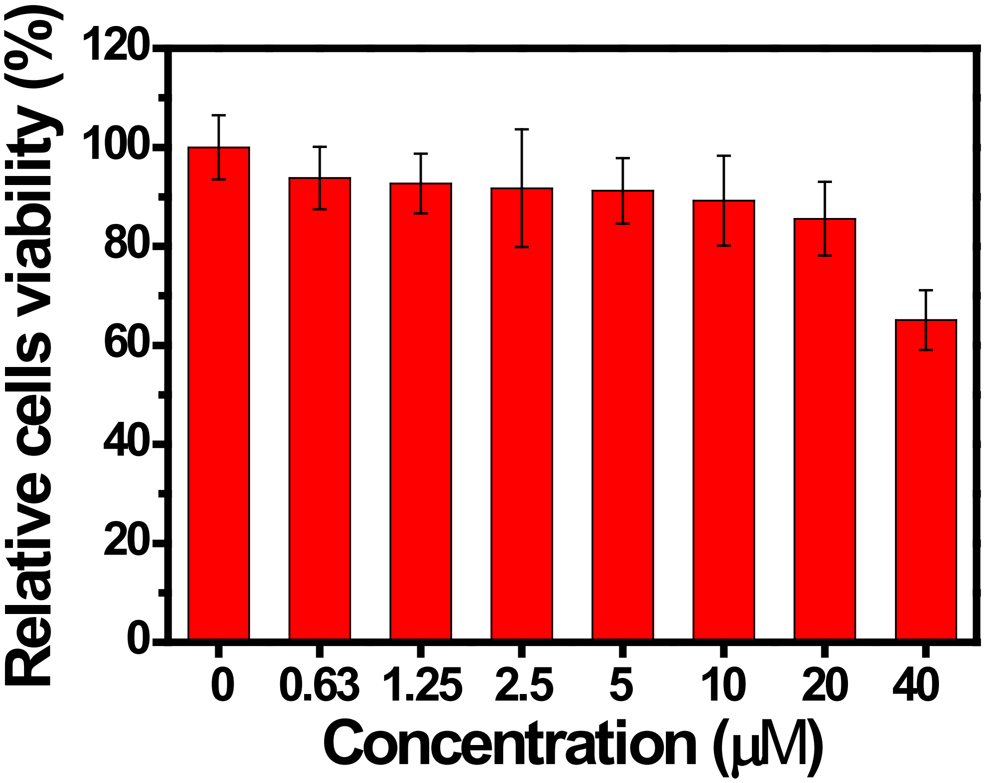

Figure S11. The relative cells viabilities of breast cancer (4T1) cells incubating with Pa-PEG nanodots for 24 h without laser irradiation.

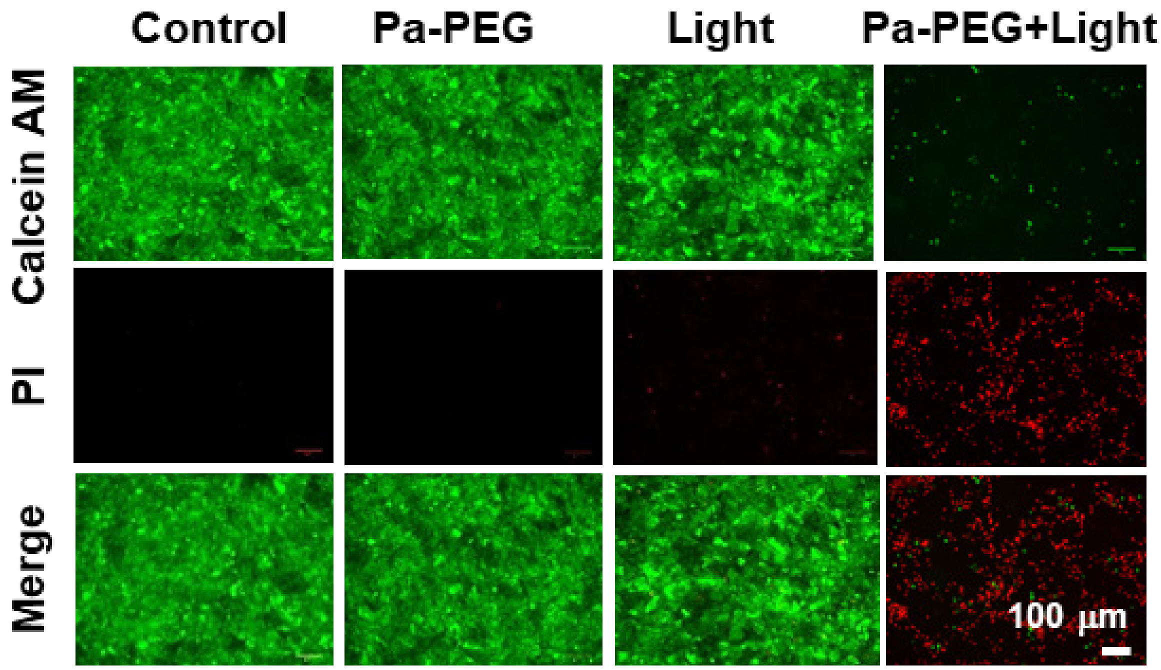

Figure S12. Fluorescence images of calcein-AM/PI co-stained 4T1 cells after various treatments with NIR-triggered light. Red and green indicated dead cells and living cells, respectively.

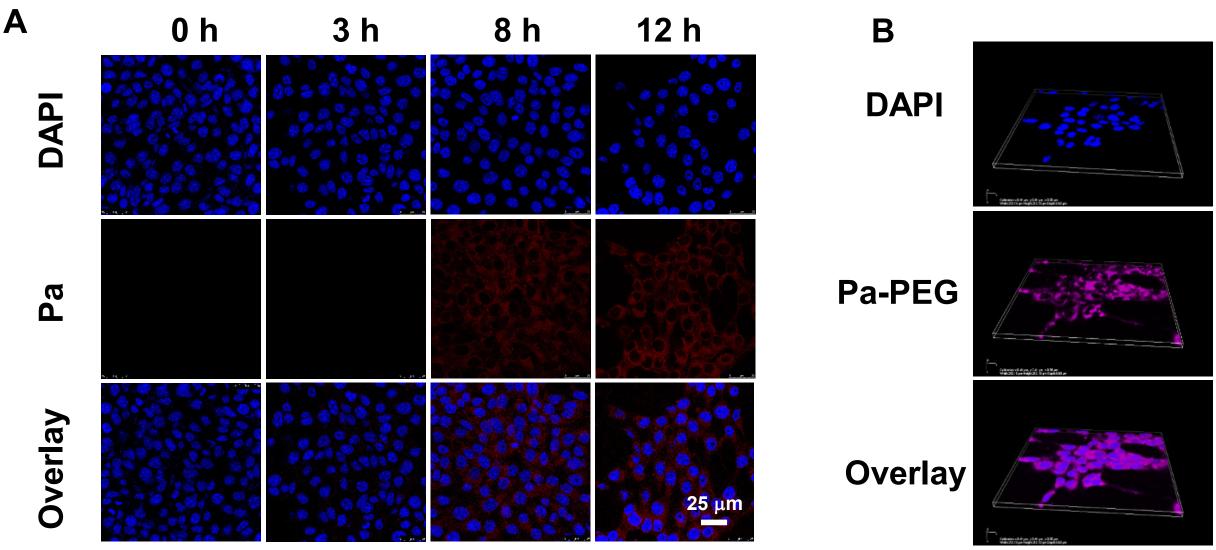

Figure S13. Confocal imaging. (A) Cellular uptake of free Pa in 4T1 cells at 0, 3, 8, and 12 h. Scale bars = 25 µm. (B) Z-stacking imaging of Pa-PEG nanodots incubated with 4T1 cells at 5 µM.

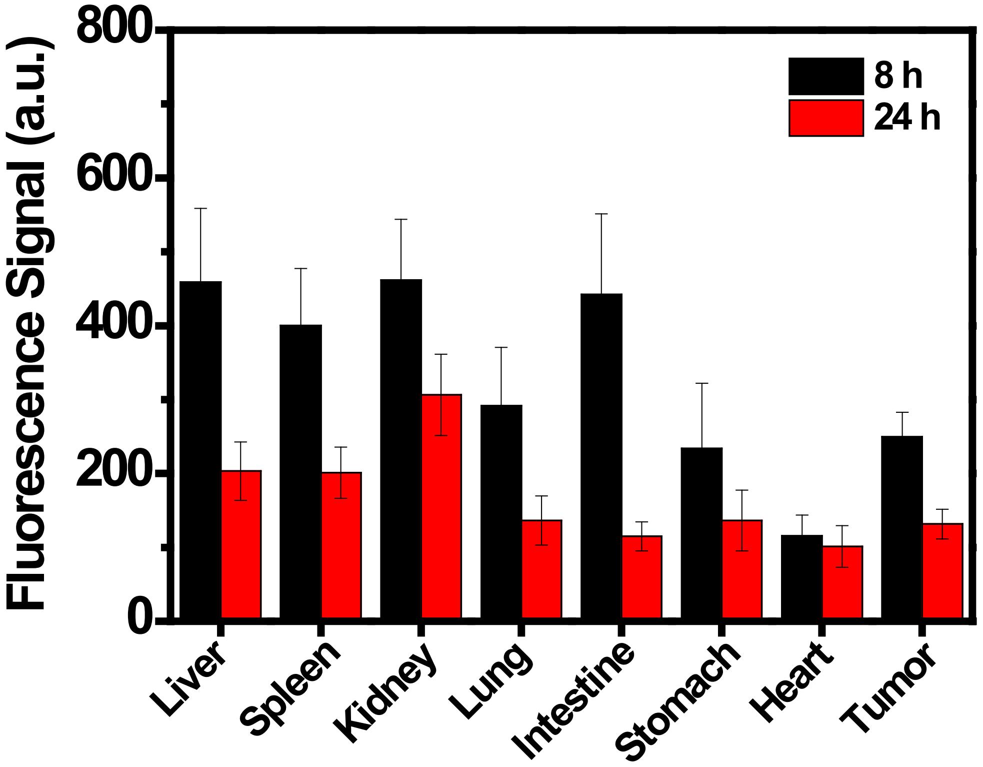

Figure S14. Fluorescence biodistribution of major organs and tumor at 8 and 24 h post injecting of Pa-PEG nanodots.

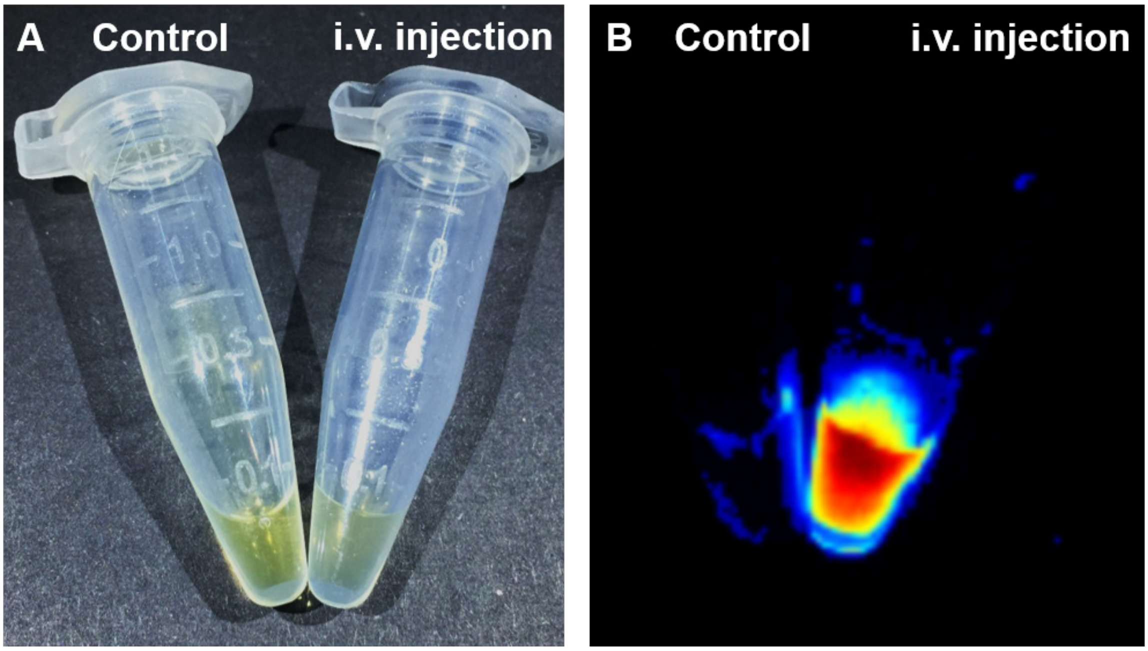

Figure S15. Photos of urine collected before and after i.v. injection of Pa-PEG nanodots for half an hour. (A) White image. (B) Fluorescent image.

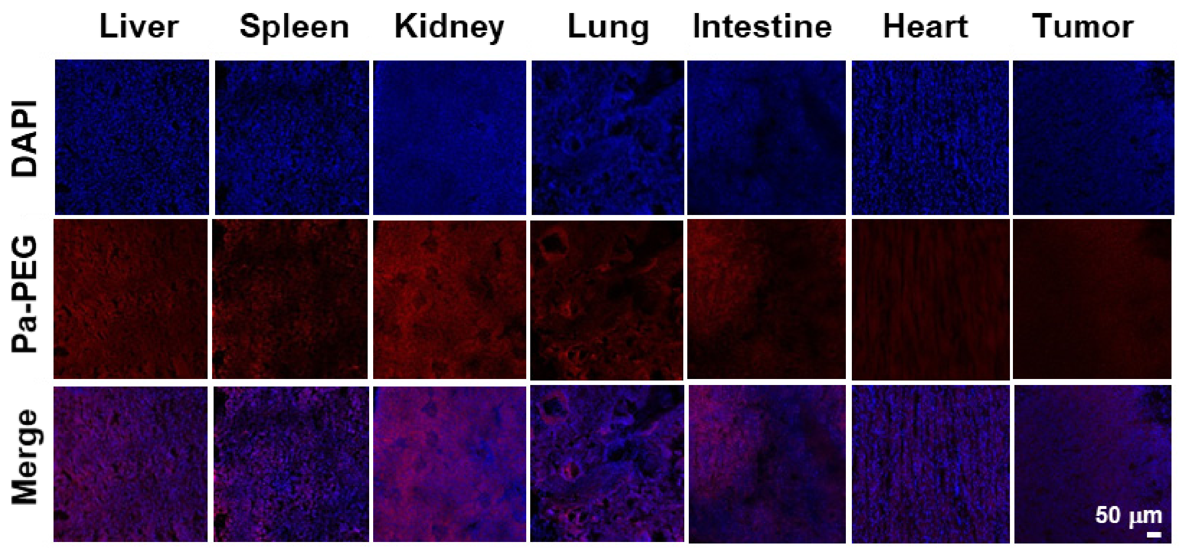

Figure S16. Confocal imaged tissue slides of major organs include liver, spleen, kidney, lung, intestine, heart, and tumor at 8 h post injection. Scale bar = 50 μm.

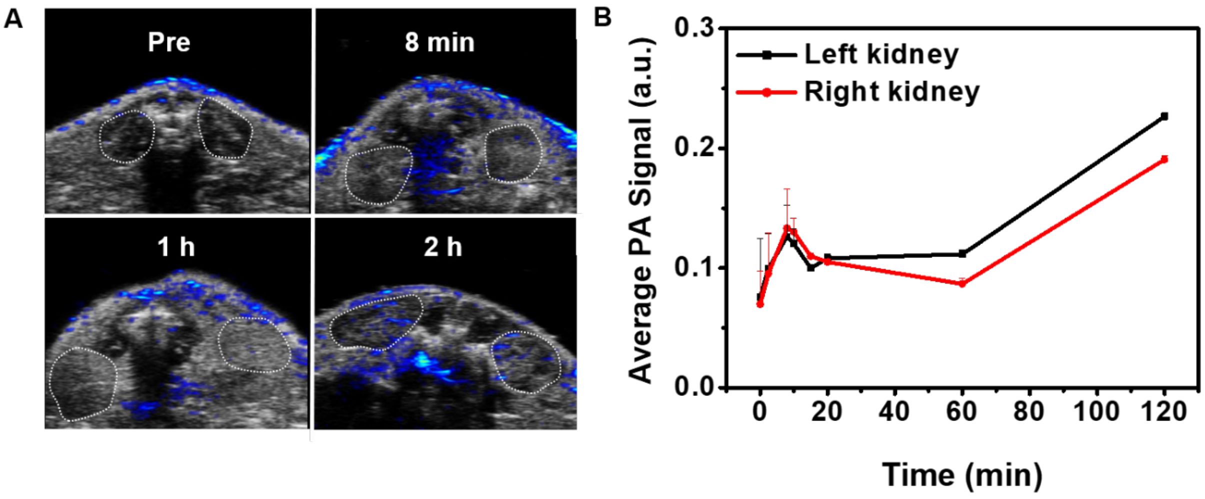

Figure S17. *In vivo* renal excretable behavior studies of Pa-PEG nanodots. (A) Real time PA signal investigation of kidney uptake at first 2 h post injecting Pa-PEG nanodots. (B) Real time PA signal monitoring in the kidney of breathing mice post injection at first 2 h.

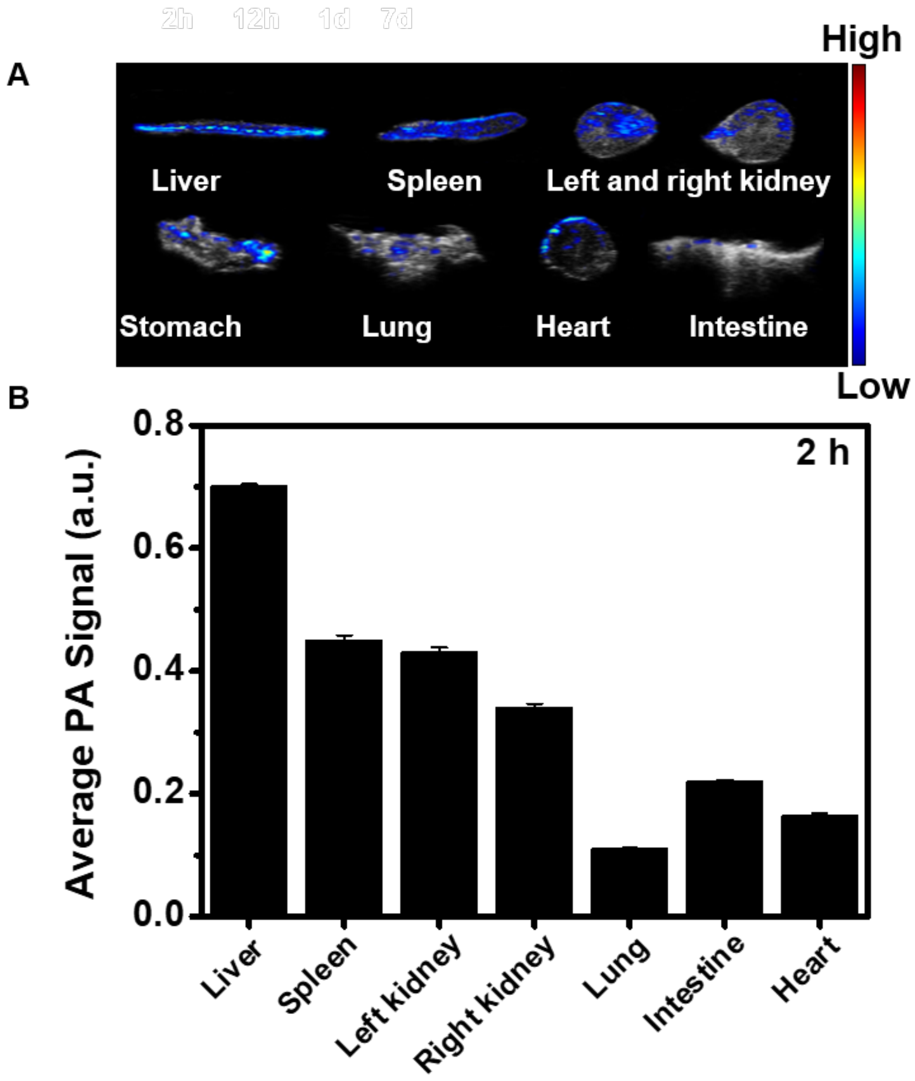

Figure S18. (A) *Ex vivo* PA images of main organs were observed 2 h p.i. (B) Photoacoustic biodistribution of main organs measured at 2 h post injecting of Pa-PEG nanodots.

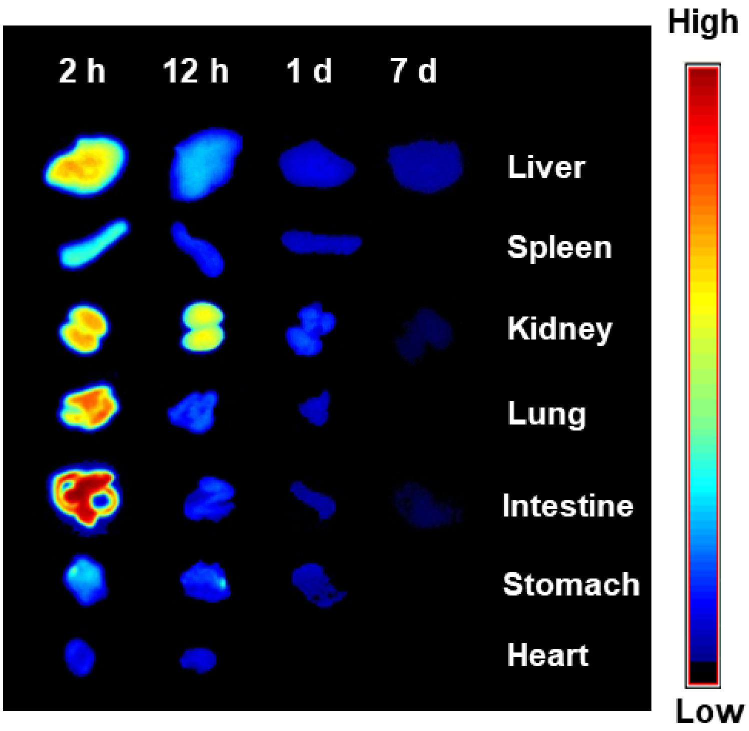

Figure S19. The obvious decreasing of *ex vivo* fluorescence of major organs over 2 h, 12 h, 1 day, and 7 days.

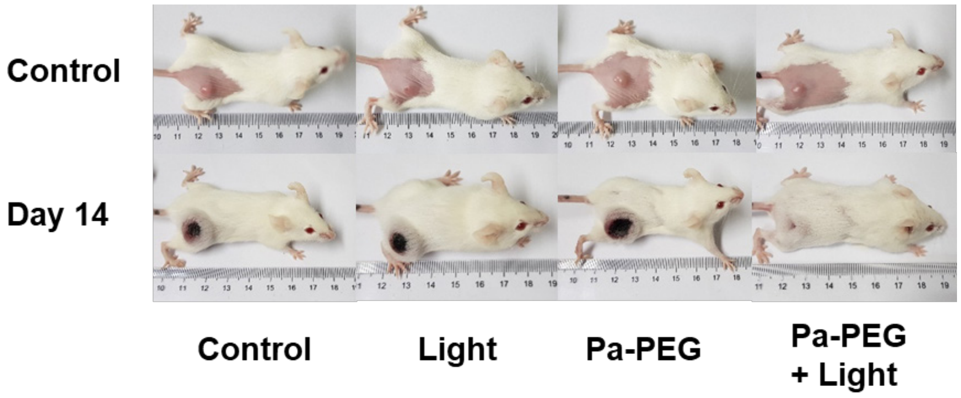

Figure S20. Representative photographs of mice from different groups taken before (Day 0) and after treatment at 14 days.

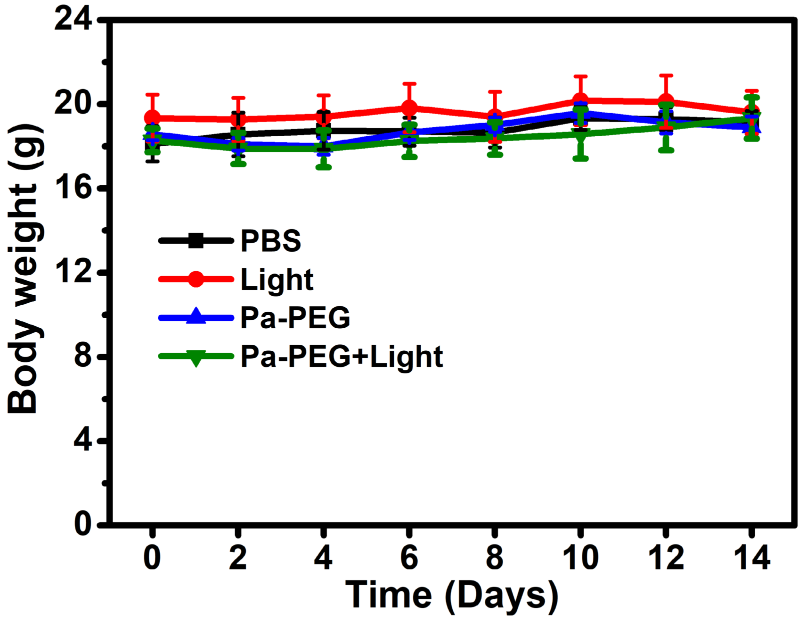

Figure S21. The body weight curves of each groups measured every other day for 14 days.
